# Orientation of Gp96 and Calreticulin T-cell epitopes in a multiepitope HPV16 E7 vaccine construct affects predicted immunostimulatory properties: An *in silico* and expression validation study

**DOI:** 10.1371/journal.pone.0353860

**Published:** 2026-07-24

**Authors:** Giti Esmail Nia, Zahra Shahosseini, Elahe Akbari, Hadi Bamehr, Fatemeh Heidarnejad, Monireh Mohsenzadegan, Azam Bolhassani, Mohammadmorad Farajollahi

**Affiliations:** 1 Department of Medical Biotechnology, School of Allied Medical Sciences, Iran University of Medical Sciences (IUMS), Hemmat Highway, Tehran, Iran; 2 Department of Basic Oncology, Health Institute of Ege University, Izmir, Turkey; 3 Department of Molecular Virology, Pasteur Institute of Iran, Tehran, Iran; 4 Department of Hepatitis, AIDS and Blood-borne Diseases, Pasteur Institute of Iran, Tehran, Iran; 5 Department of Medical Biotechnology, Biotechnology Research Center, Pasteur Institute of Iran, Tehran, Iran; 6 Department of Medical Laboratory Sciences, Faculty of Allied Medical Sciences, Iran University of Medical Sciences, Tehran, Iran; Instituto Butantan, BRAZIL

## Abstract

Heat shock proteins (HSPs) can be used as adjuvants to develop therapeutic vaccines, as they enhance the cross-presentation of related tumor antigens and stimulate T cells. In this study, the immunostimulatory properties of gp96 and calreticulin was evaluated to enhance HPV16 E7-based vaccines effectiveness. *In silico* evaluation was performed to select epitopes from HSPs based on high binding affinity to MHC-I/II, strong immunogenicity, and population coverage. Six novel multiepitope constructs harboring conserved epitopes of E7, gp96, and calreticulin were designed in different orientations. Molecular docking was performed between these constructs and signaling (TLRs) and endocytic (CD14, CD91, LOX-1, and SREC-1) receptors. After determination of an effective construct in molecular docking and MD simulation, prokaryotic expression plasmid containing eight (CTL/HTL) epitopes from HPV16 E7, gp96, and calreticulin in suitable orientation was prepared, and the recombinant multiepitope peptide was generated in *E. coli* system. Our data showed that the gp96-CRT-E7 construct had superior docking scores with receptors (especially TLR2 and LOX-1), suggesting stronger stimulation of both innate and adaptive immunity than other constructs. It was predicted to be non-toxic, non-allergenic, antigenic, immunogenic, and structurally stable. Moreover, the multiepitope gp96-CRT-E7 fusion peptide was expressed in the Rosetta strain under conditions of OD_600_: 0.6–0.7, 0.5 mM IPTG, temperature of 18ºC, and 24 hours after IPTG induction, and purified through affinity chromatography under native conditions. Generally, successful results of *in silico* and expression validation of the multiepitope gp96-CRT-E7 fusion peptide showed its strong potential as a novel candidate for HPV16 vaccine development.

## 1. Introduction

Therapeutic vaccines targeting HPV-associated cancers must trigger specific cellular immune responses to tumor antigens [1]. Heat shock proteins (HSPs) are crucial chaperone proteins involved in stress responses, conserved across species. They are produced in reaction to various stimuli and are being explored as potential therapeutics. HSPs significantly influence the immune system cells and improving the antigen presentation of tumor-associated antigens (TAA) [2,3]. They are protective of cells molecules which interact with an array of proteins to maintain cellular homeostasis and can act as natural adjuvants by enhancing the uptake of tumor antigens by antigen-presenting cells (APCs) and stimulating cytotoxic T lymphocytes (CTLs). The exceptionally high adjuvant properties of HSPs is driven via receptors for HSPs that are expressed by APCs. Generally, HSPs-based vaccines have superior application prospects and more potent immunological benefits in tumor therapy as compared to conventional tumor vaccinations [4–6]. HSPs activate the innate immune system and enhance presentation of tumor antigens to adaptive immune cells, thereby evoking synergic antitumor immunity. Consequently, they have been evaluated in new anticancer strategies such as vaccines [3,7]. Gp96 (a member of the HSP90 family) and calreticulin (CRT), two heat shock proteins, have been identified as effective immunological adjuvants that bind to several receptors on APCs such as CD91, LOX-1, and SREC-1 for antigen cross-presentation in tumor immunity among HSCs [8–10]. Calreticulin acts as an immunologic adjuvant by translocating to the cell surface with tumor-associated antigens (TAAs) and promoting tumor-specific immune responses, playing a key role in antigen recognition during cancer therapy [9]. Additionally, professional APC express CD91 that binds to gp96 and mediates the uptake of gp96–peptide complexes for optimal antigen presentation, which provides a rationale to develop therapeutic vaccines [5,10].

Computational tools allow rational design of multiepitope constructs that can be experimentally validated, accelerating the development of novel vaccine candidates [11]. Furthermore, computational biology allows scientists to precisely examine biomolecule characteristics and methods of action. The main techniques commonly employed in computational biology include pharmacophore, molecular docking, and molecular dynamics simulation [12].

While HSP-based vaccines including either calreticulin or gp96 have shown promise in preclinical studies [13–15], the potential synergistic effect of combining epitopes from both adjuvants within a single construct targeting HPV16 E7 has not been systematically investigated. The HPV16 E7 oncoprotein is an ideal antigen target due to its constitutive expression in HPV-associated cancers and its role in malignant transformation [16,17]. However, the optimal spatial arrangement of adjuvant and antigen epitopes within a multiepitope construct, and how this orientation affects immunostimulatory properties remain unexplored. To address this gap, we designed six multiepitope constructs incorporating conserved T-cell epitopes from HPV16 E7, gp96, and calreticulin in different orientations. To our knowledge, this is the first study to integrate both gp96- and calreticulin-derived epitopes in a single HPV-targeted construct with systematic evaluation of orientation effects on predicted immunogenicity.

The specific objectives of this study were to: (1) identify immunogenic T-cell epitopes from gp96 and calreticulin with high MHC binding affinity and broad population coverage; (2) design six multiepitope constructs with different epitope orientations and evaluate their interactions with immune receptors; (3) select an optimal construct based on docking scores and structural stability; (4) compare the selected multiepitope construct with its full-length counterpart; and (5) experimentally validate the expression of the final candidate in *E. coli*.

## 2. Materials and methods

### 2.1. General overview of the study

This study employed a multi-step approach: (1) identification and selection of immunogenic T-cell epitopes from gp96 and calreticulin; (2) design of six multiepitope constructs with different orientations of gp96, CRT, and E7 epitopes; (3) selection of the optimal construct through molecular docking with immune receptors; (4) comparative immunoinformatics analysis between the selected multiepitope construct and its full-length counterpart; (5) molecular dynamics simulation to assess complex stability; and (6) experimental validation through cloning and expression in *E. coli*. **A detailed description of the materials and methods used in this study is provided in** S1 File.

### 2.2. Epitope prediction of gp96 and calreticulin

The reference protein sequences for human gp96 (UniProt: P14625) and calreticulin (UniProt: P27797) were gained from the UniProt database. CTL epitopes (8–11 amino acids) were predicted by NetMHCpan 4.0 with binding affinity thresholds at the 0.5th percentile for strong binders and 2nd percentile for weak binders. HTL epitopes (14–16 amino acids) were predicted using NetMHCIIpan 4.0 with thresholds at 2% for strong binders and 10% for weak binders.

MHC-I immunogenicity was evaluated for selected epitopes utilizing the IEDB Class I Immunogenicity tool, and antigen processing (proteasomal cleavage and TAP transport) were measured using the IEDB processing prediction server. Population coverage was estimated using the IEDB population coverage tool. The IEDB conservancy analysis tool was used to analyze the cross-HPV subtype epitope conservancy. Antigenicity was determined by VaxiJen v2. 0 (threshold > 0.4), allergenicity was calculated following the criteria with AllergenFP v.1 0, and toxicity using ToxinPred. Cytokine induction (IFN-γ, IL-4, IL-10) was predicted using IFNepitope, IL4Pred, and IL10Pred servers, respectively. Antibody-specific epitopes were identified using IgPred, and linear B-cell epitopes were predicted using BepiPred-2.0.

### 2.3. Peptide-MHC molecular docking

Flexible molecular docking between selected epitopes and MHC alleles was performed using ClusPro 2.0. PDB structures for MHC class I alleles (HLA-B27:05 [1OGT], HLA-A24:02 [5HGA], HLA-A02:01 [4UQ3], HLA-A03:01 [3RL2], HLA-B35:01 [3LKN], HLA-A11:01 [1X7Q], HLA-B08:01 [3SPV], HLA-B07:02 [5EO1]) and MHC class II alleles (HLA-DRB1:0101 [4AH2], HLA-DRB1:0301 [2Q6W], HLA-DRB1:0401 [5LAX], HLA-DRB1:1101 [6CPL], HLA-DRB5:0101 [1H15]) were obtained from the RCSB database.

### 2.4. Multiepitope construct design

Based on comprehensive epitope analyses, four high-scoring epitopes (two from gp96: 192–208 and 378–389; two from calreticulin: 147–173 and 164–185) were selected and linked to previously characterized HPV16 E7 epitopes(49-57, 43-52, 71-79, and 7-15) [18]. Epitopes were connected using AAY proteolytic linkers. Six constructs with different orientations were designed using SnapGene® 3.2.1: gp96-E7-CRT, E7-gp96-CRT, gp96-CRT-E7, CRT-E7-gp96, CRT-gp96-E7, and E7-CRT-gp96.

### 2.5. Tertiary structure prediction and refinement

Three-dimensional structures of all six constructs were predicted using the I-TASSER server. The top models (based on C-score) were refined using GalaxyRefine. Refined structures were validated using ERRAT (overall quality factor >50% indicates high quality), PROCHECK (Ramachandran plot analysis), and Verify3D (≥70% residues with score >0.1)

### 2.6. Protein-protein docking with immune receptors

Molecular docking between each multiepitope construct (as ligand) and toll-like receptors (TLR2 [2Z7X], TLR3 [1ZIW], TLR4 [3FXI], TLR5 [3J0A], TLR8 [3W3G], TLR9 [3WPB]) and endocytic receptors (LOX-1, SREC-1, CD91, CD14) was performed using ClusPro 2.0. The construct with the most favorable docking scores was selected as the final candidate for further analysis.

### 2.7. Comparative analysis with full-length construct

The full-length protein sequence comprising complete gp96, calreticulin, and E7 proteins was assembled for comparison with the selected multiepitope construct. Both constructs were evaluated for: Physicochemical properties: Molecular weight, theoretical pI, amino acid composition, instability index, and half-life using ProtParam; solubility using Protein-Sol; allergenicity using AllerTOP 2.0.Secondary structure: Predicted using PSIPRED 4.0 and RaptorX.B-cell epitopes: Linear epitopes predicted using BepiPred; discontinuous epitopes predicted using Ellipro.Disulfide bonds: Predicted using DIpro scratch protein predictor. *In silico* cloning: Codon optimization for *E. coli* using JCat; CAI and GC content analysis; cloning simulation in pET-24a(+) vector using SnapGene.

### 2.8. Immune simulation

The immunogenicity and immune response profile of the final multiepitope construct were predicted using the C-ImmSim server. Simulations used homozygous host haplotypes (HLA-A0101, HLA-A0201, HLA-B0702, HLA-DRB10101, HLA-DRB1*0401) with three vaccine doses administered at days 1 (priming), 84 (first booster), and 100 (second booster). This immunization schedule was selected based on typical prime-boost regimens used in therapeutic HPV vaccine studies [15,18] and a standard protocol within the C-ImmSim simulation environment.

### 2.9. Normal mode analysis

The iMODS server was used to evaluate the movement and stability of the final construct complexed with key receptors (TLR2, TLR4, LOX-1, SREC-1). Parameters analyzed included deformability, B-factors, eigenvalues, covariance maps, and elastic networks.

### 2.10. Molecular dynamics simulation

Simulations using Gromacs 2021.5 with the CHARMM-36 force field were done to obtain trajectories lasting 50 ns for each of the constructs complexed with TLR2, TLR4, LOX-1, and SREC-1. The systems were prepared by solvating in TIP3P water, neutralizing with ions, and equilibrating in both NVT and NPT simulations.

### 2.11. Experimental expression validation

#### 2.11.1. Construction of expression plasmid.

The gp96-CRT-E7 gene was optimized and constructed (Shine Gene, China) in vector pUC57. Subcloning of the insert to pET-24a (+) was performed via restriction sites *Sal*I/*Hind*III. Confirmation of recombinant plasmids was done by PCR, restriction digestion, and sequencing of selected colonies.

#### 2.11.2. Protein expression.

Transformed BL21 (DE3) and Rosetta *E. coli* strains harboring pET-24a (+)-gp96-CRT-E7 were used for expression induction. Cultures were grown at optimal temperature 18°C or 37°C, and expression was induced by addition of 0.5 mM IPTG at OD600 = 0.6–0.7. Induction time of 4 or 24 hours was applied. Expression of the target protein was confirmed by SDS-PAGE 12.5% and Western blotting with anti-His tag antibody (Abcam, 1:10,000 *v/v*).

#### 2.11.3. Protein purification.

Cells were harvested, lysed by sonication, and subjected to centrifugation. The soluble fraction was purified with native HisPur Ni-NTA affinity chromatography. Protein was dialyzed in PBS, and protein concentration was determined by Bradford assay. Endotoxin levels were determined using LAL assay (< 0.5 EU/mg; QCL-1000).

### 2.12. Ethics approval

Not applicable. This article does not contain any studies with human participants or animals performed by any of the authors.

## 3. Results

### 3.1. Epitope selection from gp96 and calreticulin

Initial screening identified 19 CTL epitopes (11 from calreticulin, 8 from gp96) with high MHC-I binding affinity (Table 1). From these, two CTL epitopes from each protein were selected based on superior MHC-I processing scores, immunogenicity, and non-allergenic/non-toxic profiles. The selected epitopes were calreticulin 164–185 and 147–173, and gp96 378–389 and 270–285. (Detailed MHC-I processing scores, immunogenisity antigenicity, allergenicity and toxicity data for all CTL epitopes are provided in **S2 File**. Supplementary Tables(1-2).

**Table 1:** Selected CTL epitopes of calreticulin and gp96.

Protein Name	Position	Epitope Sequence	Number of binding HLA	Peptide length	Average Rank Scores*	Population coverage(world)	Conservancy
	0-22	MLLSVPLLLGLLGLAVAEPAVY	22	17	1.234	93.64	100%
	14-27	AVAEPAVYFKEQFL	22	14	0.796	96.41	100%
	66-80	QTSQDARFYALSASF	26	15	0.78	92.91	100%
Calreticulin	78-97	ASFEPFSNKGQTLVVQFTVK	23	20	0.86	96.08	100%
	107-129	GYVKLFPNSLDQTDMHGDSEYNI	22	23	0.8	94.51	100%
	147-173	FNYKGKNVLINKDIRCKDDEFTHLYTL	27	27	0.71	97.01	100%
	164-185	DDEFTHLYTLIVRPDNTYEVKI	30	22	0.5	97.78	100%
	223-266	KIDDPTDSKPEDWDKPEHIPDPDAKKPEDW	18	29	0.88	80.52	100%
	260-289	WEPPVIQNPEYKGEWKPRQIDNPDYKGTWI	18	30	0.68	74.98	100%
	295-307	NPEYSPDPSIYAY	15	13	0.73	74.62	100%
	303-318	SIYAYDNFGVLGLDLW	16	16	0.89	82.43	100%
Gp96	88-114	IINSLYKNKEIFLRELI	11	17	0.62152	73.14	100%
	192-208	GQFGVGFYSAFLVADKV	12	17	0.97701	75.73	100%
	257-273	YLELDTIKNLVKKYSQFINFPIY	19	23	0.75	94.23%	100%
	270-285	YSQFINFPIYVWSSKT	19	16	0.9455	93.97	100%
	378-389	GEVTFKSILFVPTSAPRGLFDEY	18	23	0.6526	87.78	100%
	420-435	DFHDMMPKYLNFVKGV	18	16	0.97	90.13	100%
	561-576	YEVIYLTEPVDEYCIQ	10	16	0.6871	75.27	100%
	738-753	IERMLRLSLNIDPDAK	15	16	0.6554	85.04	100%

*Lower rates show better binding affinity

#### 3.1.1. HTL epitope prediction.

HTL epitope prediction identified nine epitopes (three from calreticulin, six from gp96). The selected epitopes overlapped with CTL epitopes to enable balanced CD4⁺ and CD8 ⁺ T-cell responses. The overlapping epitopes calreticulin 147–173 and 164–185, and gp96 378–389 demonstrated the most favorable profiles (Table 2). (Complete HTL epitope data, including antigenicity, cytotoxicity and allergenicity is provided in **S2 File****.)** Supplementary Table 3)

**Table 2:** Selected HTL epitopes of calreticulin and gp96.

Protein Name	Position	Epitope Sequence	Number of binding HLA	Peptide length	Average Rank Scores*	Population coverage(world)	Conservancy
	107-129	GYVKLFPNSLDQTDMHGDSEYNI	7	23	6.75	89.23%	100%
Calreticulin	147-173	FNYKGKNVLINKDIRCKDDEFTHLYTL	7	27	5.2	88.54%	100%
	164-185	DDEFTHLYTLIVRPDNTYEVKI	10	22	5.18	84.67%	100%
Gp96	192-213	IINSLYKNKEIFLRELI IVTSK	13	22	4.8	73.23	100%
	257-273	YLELDTIKNLVKKYSQFINFPIY	7	23	4.43	60.45%	100%
	378-389	GEVTFKSILFVPTSAPRGLFDEY	11	23	3.99	52.46%	100%
	738-753	IERMLRLSLNIDPDAK	7	16	5.96	61.24%	100%
	83-98	NRMMKLIINSLYKNKE	12	16	2.7	62.46	100%
	535-549	QDKIYFMAGSSRKEA	10	15	4.3	89.82%	100%

*Lower rates show better binding affinity

#### 3.1.2. Cytokine production and antibody-specific epitopes.

The cytokine profiles indicated that peptides gp96 378–389 and calreticulin 147–173 could induce IFN-γ, IL-4, and IL-10 responses, demonstrating a possible role in balanced Th1 and Th2 reactions. Epitopes for IgG antibodies were recognized in gp96 378–389 and calreticulin **S2 File**. Supplementary Tables(4-5)

#### 3.1.3. Linear B-cell epitopes prediction.

The highest number of linear B cell epitope residues (19 residues) was found in CRT (147–173) **S2 File**. Supplementary Table 6

**3.1.4.5 Molecular docking between the selected epitopes and MHC molecules.** Molecular docking between selected epitopes and MHC molecules confirmed stable interactions, with the most favorable scores observed for gp96 378–389/HLA-B:0801 (−921.3) and calreticulin 164–185/HLA-DRB5:0101 (−910.9) (**S2 File** Supplementary Table 7).

**S1 Fig** indicates examples of successful peptide-protein docking between the selected MHC class I molecules and gp96 and calreticulin epitopes.

### 3.2. Design of multiepitope construct based on immunoinformatic analyses

Based on our previous findings [18], we identified a range of unique features in the selected HPV16 E7 epitopes, which have set the stage for further analyses. The identified E7 epitopes were antigenic, non-toxic and non-allergenic, and they had an ability to activate B cells and IFN-γ secretion(**S2 File**. Supplementary Table 8).

Six constructs with different epitope orientations were designed using AAY linkers: gp96-E7-CRT, E7-gp96-CRT, gp96-CRT-E7, CRT-E7-gp96, CRT-gp96-E7, and E7-CRT-gp96. four high-scoring CTL and HTL overlapping epitopes (two epitopes of 192–208 and 378–389 from gp96, and two epitopes of 164–185 and 147–173 from calreticulin), along with three CTL epitopes of 49–57, 43–52 and 71–79 and one HTL epitope of 7–15 from HPV-16 E7.

### 3.3. Selection of final multiepitope construct

#### 3.3.1. Prediction, refinement, and validation of tertiary structures.

I-TASSER generated five 3D models for each construct. The gp96-CRT-E7 construct had the most favorable C-score (−1.55). Following refinement with GalaxyRefine, all structures showed >90% of residues in favored/additionally allowed regions of the Ramachandran plot and ERRAT overall quality factors >80%, indicating high-quality models.For stance, the optimal model’s C-score was −1.55 for the multiepitope gp96-CRT-E7 (**S2A Fig**). Each construct’s top model was improved via the Galaxy Refine server. Robetta server produced the chimeric vaccination construct’s 3D model. Over 90% of the residues in the selected area suggest sufficient stereochemical structure. Over 90% of the residues in our structures were located in the Ramachandran plot’s extra-permitted and preferred regions, indicating that these models have a high degree of amino acid conformation validity and tend to be of high quality. The findings demonstrated that all structures have an overall quality factor of greater than 80%. (**S2B Fig**).

#### 3.3.2. Protein-protein docking between TLRs/ specific receptors and different multiepitope constructs.

**Fig 1:**
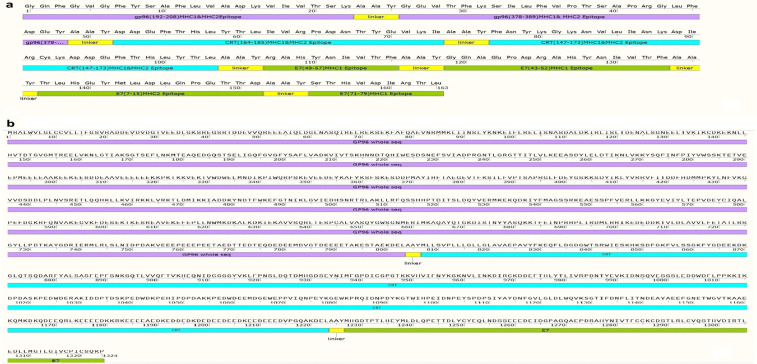
The designed gp96-CRT-E7 multiepitope construct and its corresponding whole sequence construct: (a) gp96-CRT-E7 multiepitope construct, (b) gp96-CRT-E7 whole sequence construct.

**Fig 2:**
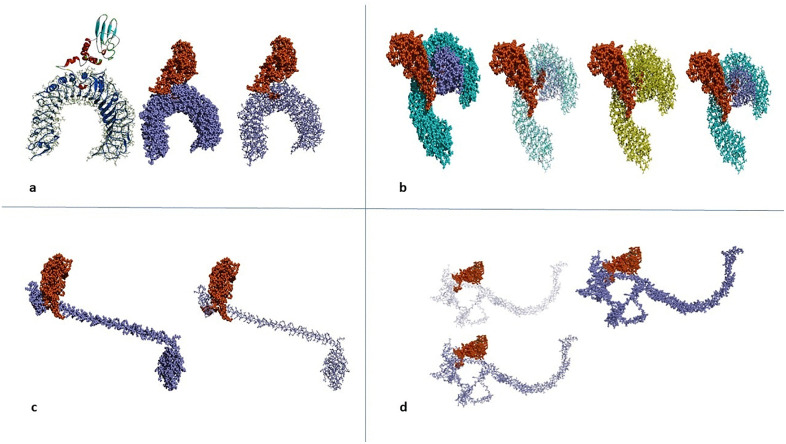
Molecular docking of the gp96-CRT-E7 multiepitope construct and main receptors: (a) TLR-2, (b) TLR-4, (c) LOX-1, (d) SREC-1; Red indicated the gp96-CRT-E7 multiepitope vaccine structure and purple and green showed receptor.

Six designed constructs were docked with TLRs (TLR2, TLR3, TLR4, TLR5, TLR8, and TLR9) and endocytic receptors (LOX-1, SREC-1, CD91, and CD14). The gp96-CRT-E7 construct had higher binding affinities among most receptors, especially with TLR2 (−1129.8), TLR4 (−1139.8), TLR5 (−1546.4), TLR9 (−1384.9), LOX-1 (−1317.8), and SREC-1 (−1220.4) (Tables 3 and 4). The LOX-1/ gp96-CRT-E7 formed the most stable complex. LOX-1 can stimulate the immune system through its interaction with SREC-I, and CD14, suggesting that the gp96-CRT-E7 multiepitope construct may enhance antigen presentation (Table 4). In docking analysis, the strongest interactions with TLRs occurred when the gp96 and CRT epitopes were linked to the N-terminal of the E7 epitopes. Based on these results, the gp96-CRT-E7 construct was selected as the final vaccine candidate for further characterization. The complete amino acid sequence of this construct is shown in Fig 1A, with its full-length in Fig 1B. Molecular docking visualizations of gp96-CRT-E7 with TLR2, TLR4, LOX-1, and SREC-1 are presented in Fig 2.

**Table 3:** Protein-Protein docking results (lowest energy in the best model) between different multiepitope constructs and TLRs.

Multiepitope Constructs	TLR2 Docking score	TLR3 Docking score	TLR4 Docking score	TLR5 Docking score	TLR8 Docking score	TLR9 Docking score
E7-CRT-gp96	−850.8	−1070.7	−880.8	−1244.3	−958.8	−1224.1
E7-gp96-CRT	−942.8	−868.5	−940.2	−1292.4	−953	−1117.4
CRT-gp96-E7	−984.6	−983.9	−1150.7	−1408.2	−1433.6	−1322.3
CRT-E7-gp96	−871.2	−838.1	−945.5	−1238.6	−883.6	−1367.1
gp96-CRT-E7	−1129.8	−870.8	−1139.8	−1546.4	−1257.9	−1384.9
gp96-E7-CRT	−964	−1174.1	−961.6	−1359.7	−1031.9	−1272.7

**Table 4:** Protein-Protein docking results (lowest energy in the best model) between different multiepitope constructs and specific receptors.

Multiepitope constructs	SREC-1 Docking Score	LOX-1 Docking Score	CD91 Docking Score	CD14 Docking Score
E7-CRT-gp96	−997.9	−968.4	−671.9	−1244.3
E7-gp96-CRT	−1220.3	−1045.5	−724.7	−989.3
CRT-gp96-E7	−1080.9	−1291.0	−925.3	−953.3
CRT-E7-gp96	−1223.2	−1110.5	−856.6	−940.6
gp96-CRT-E7	−1220.4	−1317.8	−932.7	−989.9
gp96-E7-CRT	−1284.8	−1066.5	−776.0	−863.6

### 3.4. Comparative analyses of the gp96-CRT-E7 multiepitope construct with full-length construct

#### 3.4.1. Physicochemical features.

The multiepitope gp96-CRT-E7 construct (18.57 kDa) was substantially smaller than its full-length counterpart (152.21 kDa), meeting the recommended <110 kDa criterion for vaccine development [19]; thus our final gp96-CRT-E7 multiepitope construct with a molecular weight of 18.57 kDa is suitable for further development. Both multiepitope and full-length gp96-CRT-E7 constructs had a half-life of 30 hours. The designed gp96-CRT-E7 multiepitope construct was stable, indicating its sufficient and favorable stability, whereas its full-length sequence was unstable (Table 5). The multiepitope construct was predicted to be stable (instability index: 24.16), while the full-length construct was unstable (44.88). Both constructs were soluble (solubility scores >0.45), non-allergenic, and antigenic (VaxiJen scores >0.4) (Table 5). In both multiepitope and full-length gp96-CRT-E7 constructs, the number of negatively charged residues is more than that of positively charged ones (*i.e.,* 20 negative versus 14 positive residues in the multiepitope construct and 299 negative versus 171 positive residues in the full-length construct) indicating an overall acidic character for both proteins. This high negative charge can be advantageous in aspects of increasing solubility and hydrophilicity, which is valuable for vaccine formulation. The pI of the multiepitope construct (5.41) was more neutral than that of the pI of the full-length construct (4.54), suggesting a low negative charge at physiological pH.

**Table 5:** Physicochemical properties of the multiepitope and full-length gp96-CRT-E7 constructs.

Construct	Molecular weight (kDa)	Positive charge residue	Negative charge residue	Theoretical PI	Predicted scaled instability	Stability	Half life	Solubility >0.45	antigenicity>0.4	Allergenicity
Gp96-CRT-E7 (Epitopes)	18574.90	14	20	5.41	24.16	stable	30 hours	0.671	0.5281 ANTIGEN	Non allergen
GP96-CRT-E7 (Whole sequence)	152207.38	171	299	4.54	44.88	unstable	30 hours	0.571	0.6400 ANTIGEN	Non allergen

#### 3.4.2. Secondary prediction.

PSIPRED analysis showed that the multiepitope construct comprised 52% coil, 27% α-helix, and 20% β-sheet, with 36% exposed residues. The full-length construct showed 55% coil, 28% α-helix, and 16% β-sheet, with 51% exposed residues. The multiepitope construct exhibited less disorder (1% vs. 26%) (**S3 Fig****).**

#### 3.4.3. B-cell epitope prediction.

The multiepitope construct contained seven linear B-cell epitopes (scores 0.511–0.869) and five discontinuous epitopes (scores 0.638–0.828), indicating potential for humoral immune activation.

(Predicted linear and discontinues B-cell epitopes of the multiepitope gp96-CRT-E7 construct are presented in **S2 File**. Supplementary Tables 9-10. (**S4 Fig****).**

#### 3.4.4. Predicting disulfide-bonding state.

The multiepitope construct contained four cysteines, insufficient for disulfide bond formation. The full-length construct had 15 cysteines predicted to form seven disulfide bonds.

### 3.5. Confirmation of the multiepitope construct

#### 3.5.1. Immune response simulation.

**Fig 3:**
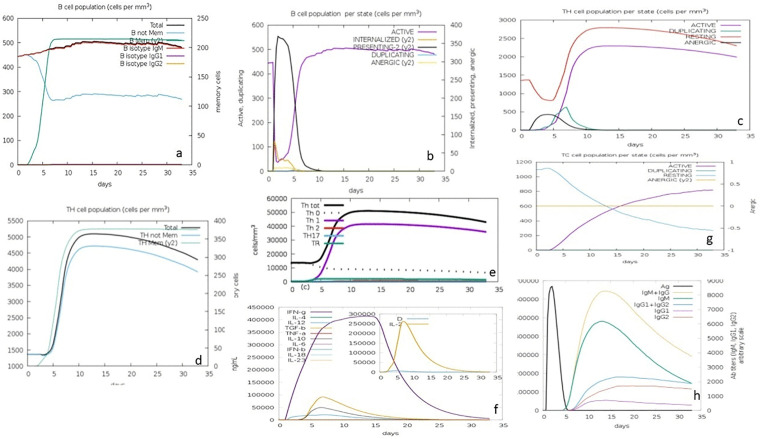
Immune simulation prediction of the gp96-CRT-E7 multiepitope construct: (a) The evolution of B-cell populations following the three injections; (b) The active B-cell population is observed with the administration of vaccine; (c) The generation of the active T helper cell population, (d) T helper cell population is observed with the administration of vaccine; (e) The generation of T cells; (f) The cytokine profile shows the levels of IFN-γ and IL-2 induced upon administration of vaccine; (g) The generation of cytotoxic T cells was found after the vaccine injection. The RESTING indicates the cells that were not shown to the antigens while ANERGIC indicates the tolerance level of antigen; (h) The immunoglobulins production represents B-cell proliferation and stimulation of immune response after the vaccine administration.

The immune system’s response to vaccination at various time points was predicted using the C-immune technique.The findings verified that the apparent immune response and actual immunological reactions such as the detection of B-cells, T-cytotoxic cells, T-helper cells, interleukin/interferon production, and the subsequent generation of antibodies were consistent. The intermittent dosing of our multiepitope gp96–CRT-E7 vaccine construct, administered three times on days 1, 84 and day100 resulting in elevated concentrations of activated B cell as well as increased levels or IgM plus combination urinary IgG + IgM antibodies (Fig 3), was found to be optimal at the lowest dose.

The multiepitope vaccine construct also strongly upregulated secretion of IFNg and IL-2 known Th1-associated cytokines, together with marked enhancement in memory T cell populations as well cytotoxic T cells (Tc) development (Fig 3d,e,f). This profile of diverse cytokines illustrates a dominance of CTL and Th1 type responses to promote effective stimulation of cellular immunity. The third administration led to further increases in memory T cells, activated T cells and total T cell levels (Fig 3c,d,g).

In contrast, the population of non-memory T cells, resting T cells, and regulatory T cells (Treg) significantly decreased, highlighting high efficacy of construct and providing reassurance about the vaccine’s potential. Conversely, the levels of IL-10 and IL-4 cytokines did not differ significantly from baseline, indicating a low Th2-type immune response. Further, production of the TH1-polarizing cytokine IL-12 was also largely unchanged due to limited activation dendritic cells and macrophages. Indeed, however modest levels of IL-12 can augment antigen presentation and may actually reinforce a positive feedback loop that augments IFN-γ production.

#### 3.5.2. Normal mode analysis of the multiepitope gp96-CRT-E7-receptor complexes.

A range of structural features was analyzed to evaluate the atomic mobility and stability of the gp96-CRT-E7 construct. The stability and dynamic behavior of the gp96-CRT-E7/ LOX-1 and gp96-CRT-E7/SREC-1 docked complexes were assessed using molecular dynamics simulations conducted through the iMODS service. Within the dynamic region, the covariance matrix illustrated the correlations between amino acid residues (**S5A Fig** **(E & J)).** Correlated residues were indicated in red, anti-correlated residues in white and uncorrelated residues in blue.

The elastic network model, which represents interactions between atoms as spring-connected pairs, identified stiffer regions of the structure through gray shading. As a result, the gp96-CRT-E7/SREC-1 complex was stiffer and structurally more stable than the gp96-CRT-E7/LOX-1 complex. The B-factor plots represent the atomic fluctuations, where lower values show more structural stability (**S5A Fig** (A & F)). The deformability graphs illustrate amino acid residues with greater mobility, signifying flexible regions within the complexes (**S5A Fig** **(B & G)).** The gp96-CRT-E7/SREC-1 complex showed better structural stability as compared to the gp96-CRT-E7/LOX-1 complex. Eigenvalues are directly proportional to the energy required for structural deformation (**S5A Fig** **(C&H)).** The gp96-CRT-E7/LOX-1 and gp96-CRT-E7/SREC-1 complexes yielded eigenvalues of 7.959 × 10^‒^⁶ and 1.485 × 10^‒^⁶, respectively indicating minimal energy requirements for structural rearrangement in the gp96-CRT-E7/SREC-1 complex. In comparison, the gp96-CRT-E7/TLR2 and gp96-CRT-E7/TLR4 complexes exhibited eigenvalues of 1.555 × 10^‒^⁶ and 1.616 × 10^‒^⁶, respectively (**S5b Fig** **(C & H))**. Indeed, the covariance maps for the gp96-CRT-E7/TLR2 and gp96-CRT-E7/TLR4 complexes visually depicted the relationships between residues, with red indicating correlated motion, white for uncorrelated, and blue for anti-correlated interactions (**S5b Fig** **(E & J)).** The elastic network maps revealed stiffer regions through darker gray areas (**S5b Fig** **(D & I)).** When we compared two complexes (gp96-CRT-E7/TLR-4 and gp96-CRT-E7/TLR-2) we found that atoms in gp96-CRT-E7/TLR-4 is far more flexible than atoms in gp96-CRT-E7/TLR-2 complex.

#### 3.5.3. Molecular Dynamics (MD) simulation of the TLR2/TLR4/SREC-1/LOX-1-multiepitope gp96-CRT-E7 complexes.

**Fig 4:**
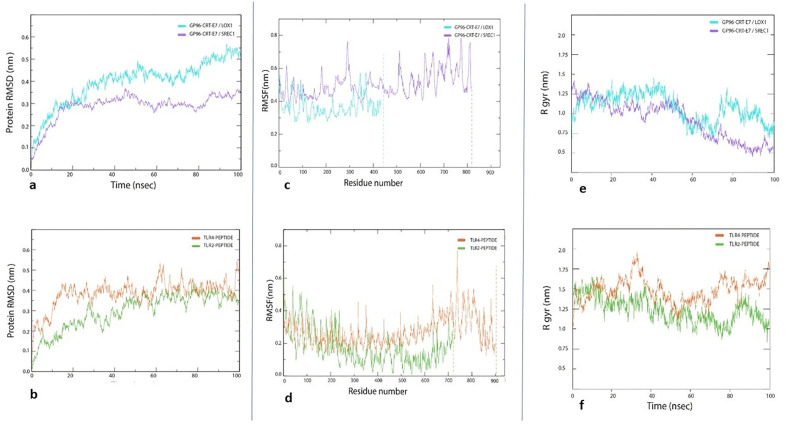
Molecular Dynamics (MD) simulation of the gp96-CRT-E7 vaccine construct/ receptor complexes: (a) Root mean square deviations (RMSD) plot of the gp96-CRT-E7 construct/ LOX-1 (blue color) and the gp96-CRT-E7 construct/SREC-1 (purple color) complexes; (b) RMSD plot of the gp96-CRT-E7 construct/ TLR4 (orange color) and the gp96-CRT-E7 construct/ TLR2 (green color) complexes; (c) Root Mean Square Fluctuation (RMSF) plot of the gp96-CRT-E7 construct/ LOX-1 (blue color) and the gp96-CRT-E7 construct/ SREC-1 (purple color) complexes; (d) RMSF plot of the gp96-CRT-E7 construct/ TLR4 (orange color) and the gp96-CRT-E7 construct/ TLR2 (green color) complexes; (e) Radius of gyration (Rg) plotted against simulation time of the gp96-CRT-E7 construct/ LOX-1 (blue color) and the gp96-CRT-E7 construct/ SREC-1 (purple color) complexes; (f) Rg plotted against simulation time of the gp96-CRT-E7 construct/ TLR4 (orange color) and the gp96-CRT-E7 construct/ TLR2 (green color) complexes.

Protein backbone stability is inversely related to the root mean square deviation (RMSD); higher RMSD values generally reflect increased structural instability. During the initial 50 ns of molecular dynamics (MD) simulations, all complexes showed a rise in RMSD due to the typical relaxation of proteins in an aqueous environment-a well-known occurrence in MD analyses. The gp96-CRT-E7/SREC-1 and gp96-CRT-E7/TLR4 complexes reached equilibrium at approximately 20 ns, whereas the gp96-CRT-E7/LOX-1 complex was stabilized around 45 ns. The gp96-CRT-E7/TLR2 complex remained consistently stable throughout the simulation (Fig 4a, b). Average RMSD values were recorded as 0.325 nm (range: 0.1–0.55 nm) for gp96-CRT-E7/SREC-1 and 0.425 nm (range: 0.5–3.5 nm) for gp96-CRT-E7/LOX-1. The gp96-CRT-E7/TLR2 and gp96-CRT-E7/TLR4 complexes showed average RMSDs of 0.445 nm (range: 0.39–0.5 nm) and 0.35 nm (range: 0.2–0.5 nm), respectively. All systems displayed RMSD averages below 0.2 nm, indicating that they achieved stable conformations during simulation. While minor variations occurred between replicate runs, all complexes demonstrated steady RMSD plateaus, suggesting that the structures oscillated around a stable mean conformation. This consistency supports the reliability of the simulations. Notably, the gp96-CRT-E7/SREC-1 complex exhibited greater stability than the gp96-CRT-E7/LOX-1 complex, as shown by RMSD data (Fig 4a, b). On the contrary, a high root mean square fluctuation (RMSF) signifies a flexible area, whereas a low RMSF suggests restricted movements throughout the MD simulation. A fluctuation value below 2Å is permissible for a small protein [20,21]. Comparison of the fluctuations between the gp96-CRT-E7/SREC-1 and gp96-CRT-E7/ LOX-1 complexes demonstrated the highest RMSF fluctuations in the regions of 595–610 and 50–90, respectively. The areas covering residues 710–800 in TLR-4 and 410–500 in TLR-2 showed notable fluctuations. The RMSF plots showed that each complex had residual fluctuations in several different areas. The gp96-CRT-E7/LOX-1 complex showed the largest amounts of dynamic variation in the areas between residues 280–300 and 700–720, excluding from the N- and C-terminal residues. Similarly, the region spanning residues 510–580 showed comparable fluctuations in the gp96-CRT-E7/SREC-1 complex. The average RMSF values were calculated as 0.6 for the gp96-CRT-E7/LOX-1 complex and 0.4 for the gp96-CRT-E7/SREC-1 complex, respectively. RMSF analysis confirmed that the overall structural integrity of complexes remained stable throughout the molecular dynamics simulation (Fig 4c, d). These findings suggest that all gp96-CRT-E7/receptors complexes possess considerable structural flexibility and stability, indicative of favorable interactions with immune receptors. In addition, to evaluate the compactness and rigidity of the complexes under study during the simulation, the radius of gyration (Rg) calculated from the molecular dynamics trajectory was used. Since lower values of radius of gyration are associated with a more stable system and greater compactness of structural units, the opposite is also true: higher values of Rg correspond to a looser and less stable arrangement of structural units. Rg analysis showed that the gP96-CRT-E7/LOX-1 complex had more radius of gyration than the one containing SREC-1. The gp96-CRT-E7/SREC-1 complex had higher structural stability. The average Rg values were determined to be approximately 1.87 Å (ranging from 0.75 to 1.5 Å) for the gp96-CRT-E7/LOX-1 complex and approximately 0.95 Å (ranging from 0.5 to 1.4 Å) for the gp96-CRT-E7/SREC-1 complex. Additionally, the TLR4/gp96-CRT-E7 complex exhibited an Rg range of 1.20 to 1.9 Å with an average of 1.55 Å, while the TLR2/gp96-CRT-E7 complex showed a range of 0.9 to 1.7 Å, averaging 1.3 Å. The Rg plots for all complexes displayed fluctuations of less than 2Å, suggesting that all of them maintained stable conformations throughout the MD simulations (Fig 4e, f).

### 3.6. *In silico* cloning of the multiepitope gp96-CRT-E7 vaccine construct

The JCat server was used to assess the Codon Adaptation Index (CAI) and GC content. The CAI and GC content for our enhanced nucleotide sequence were 1.0 and 50.11%, respectively, indicating the feasibility of effective expression in a prokaryotic system. To compare, *in silico* cloning evaluation was conducted for the complete gp96-CRT-E7 construct. The CAI and GC content of the full-length gp96-CRT-E7 construct were 0.743 and 46.0%, respectively, indicating reduced expression in the host system relative to its multiepitope construct. The multiepitope gp96-CRT-E7 gene was cloned between the restriction sites of *Sal*I and *Hind*III and the full-length gp96-CRT-E7 gene was cloned between the restriction sites of *Pae*R71 and *Eco*53kI (**S6 Fig****).**

### 3.7. Experimental validation

#### 3.7.1. Construction of the Plasmid DNA.

**Fig 5:**
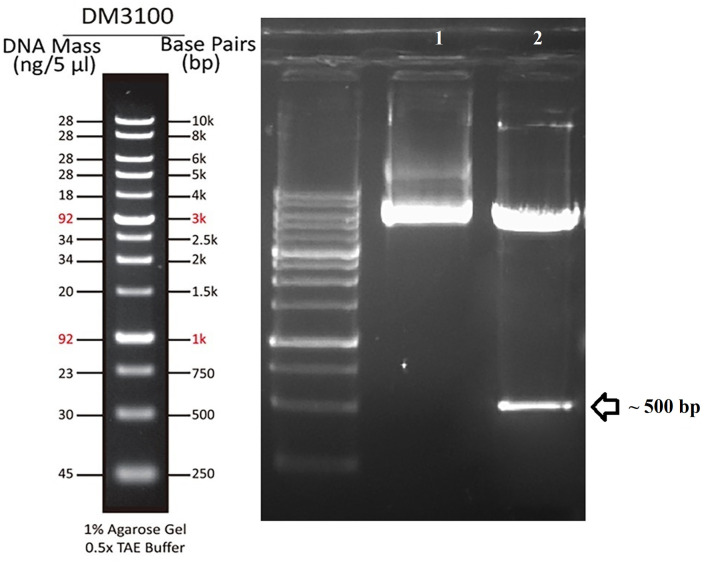
Confirmation of successful cloning in pET24a via restriction enzymes of *Sal*I and *Hind*III digestion: DNA Ladder: 1 kb (Fermentas). Lane 1: Undigested plasmid pET24a (showing a single supercoiled band; ~ 5810 bp); Lane 2: Double-digested plasmid showing two bands one at ~500 bp corresponding to the inserted fragment (gp96-CRT-E7) and one upper band representing the vector pET24a backbone (~ 5310 bp).

The gp96-CRT-E7 gene was successfully subcloned into the pET24a (+) vector. The confirmation of the recombinant pET24a-gp96-CRT-E7 was achieved through digestion and colony PCR, resulting in a distinct band of approximately 500 bp corresponding to the gp96-CRT-E7 gene on agarose gel (Fig 5).

#### 3.7.2. Expression and purification of the multiepitope gp96-CRT-E7 gene *in E.coli.*

**Fig 6:**
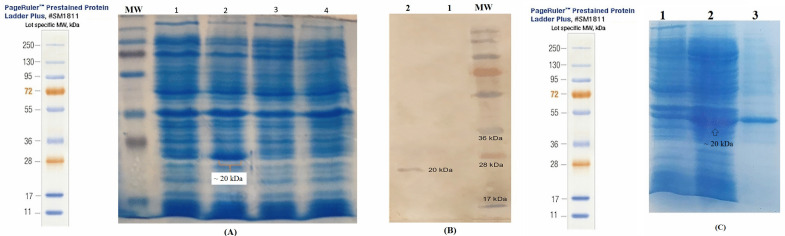
Expression and purification of the gp96-CRT-E7 multiepitope peptide in *E. coli* BL21 and Rosetta strains before and 24 h after IPTG induction at 18°C: A) SDS-PAGE analysis of the expressed protein: Lane 1: protein expression in Rosetta before IPTG induction; Lane 2: protein expression in Rosetta after IPTG induction; Lane 3: protein expression in BL21 before IPTG induction; Lane 4: protein expression in BL21 after IPTG induction; B) Western blot analysis of the expressed protein: Lane 1: protein expression in Rosetta before IPTG induction; Lane 2: protein expression in Rosetta after IPTG induction. MW is Thermo Scientific PageRuler™ Plus Prestained Protein Ladder (11–250 kDa); C) SDS-PAGE analysis of the purified protein: Lane 1: protein expression in Rosetta before IPTG induction; Lane 2: protein expression in Rosetta after IPTG induction; Lane 3: the purified protein.

The expression of the multiepitope gp96-CRT-E7 gene was performed in *E. coli* BL21 (DE3) and Rosetta under various circumstances, and confirmed using SDS-PAGE and western blotting. The results showed that the multiepitope gp96-CRT-E7 gene was expressed in Rosetta strain under conditions of OD_600_: 0.6–0.7, 0.5 mM IPTG, temperature of 18ºC and 24 h after IPTG induction as a clear band of ~ 20 kDa. The solubility test showed that the multiepitope peptide was found in soluble phase, thus it was purified using Ni-NTA affinity chromatography under native conditions and dialyzed against PBS1X (Fig 6A-C). The protein concentration was determined to be 0.320 mg/mL using the Bradford assay.

## 4. Discussion

Therapeutic vaccines targeting HPV-associated cancers must trigger a specific cellular immune response to tumor antigens [22]. HSP-TAA complexes could prove an effective formulation strategy for the development of therapeutic HPV vaccines based on DNA, protein or peptide [3,13,14,23,24] which emphasize the efficiency of HSP-E7 fusion vaccines in provoking durable antitumor immune responses.

This study presents the first systematic evaluation of epitope orientation effects in a multiepitope vaccine construct incorporating both gp96 and calreticulin as immunological adjuvants targeting HPV16 E7. Through comprehensive *in silico* analyses supported by expression validation, we identified the gp96-CRT-E7 construct as the optimal candidate with superior predicted immunostimulatory properties.

The immunostimulatory strength of HSPs may be due to their C- or N-terminal domains. Although the full-length HSPs can induce NK cell and DC proliferation, their C- or N-terminal fragments can also induce strong immunostimulatory activity [25–27]. A key finding of this study is that the arrangement of epitopes significantly affects predicted interactions with immune receptors.

Selecting overlapped CTL and HTL epitopes was aimed at enhancing T-cell response and achieving balanced immunogenicity [28]. The preferred HSP epitopes (gp96 378−389, gp96 192−208; calreticulin 147−173, calreticulin 164−185) demonstrated high MHC binding affinity, extensive population coverage (up to 97.78%), and satisfactory immunogenic properties. These findings align with previous studies identifying immunogenic regions in HSPs. Bergmann *et al*. (2021) suggested two monoclonal antibodies and suggested that 138–157, 108–127, 158–177 and 248–267 sequences of calreticulin can be considered in future therapeutic approaches [29]. These epitopes shared regional sequence similarity with our calreticulin peptides (164−185 and 147−173), although we have extended epitopes to longer peptides sequence, aiming to enhance their immunogenic potential. Additionally, Hong *et al*. investigated that a 39−272 fragment of CRT was reported to exhibit robust adjuvant properties when linked to polysaccharides or presented as a component of a fusion protein [30], which had similarity with our predicted CTL/HTL calreticulin epitopes (66−80, 78−97, 107−129, 147−173, 164−185, 223−266). Another vaccine candidate was potential for treatment of atherosclerosis through the induction of immune responses toward Th2. For calreticulin and HSP60, six new IL-4 inducers were determined [31]. This reflects how CRT epitopes have that capacity to regulate immune responses.

Our gp96-CRT-E7 construct showed even stronger binding affinities and potential for antigen presentation, indicating that adding CRT and a dual-adjuvant strategy might further increase CD8 ⁺ T-cell responses. Moreover, a chimeric DNA vaccine against HPV-16 E7 (SP-SA-E7-4–1BBL) with the endogenous signal peptide of Calreticulin showed prophylactic and therapeutic efficacy in mice with cervical cancer [15]. Based on these studies, CRT-derived peptides were considered as a promising candidate for inducing response in our multiepitope vaccine design.

On the other hand, gp96 or its N-terminal or C-terminal fragments were incorporated in several multiepitope vaccine constructs as an adjuvant. CD133 epitope vaccine with gp96 as adjuvant elicited an antitumor T cell response against leukemia [32], or in another study, liver- derived gp96-adjuvanted Influenza monovalent vaccine could broadly elicit CD8^+^ T cell immunity [10]. Zheng *et al.* (2019) demonstrated the immune adjuvant role of gp96 in DC-based vaccines, wherein it would also act as an antigen source [33]. Padula *et al.* (2023) studied the gp96-Ig vaccine platform, which combines antigen specificity and adjuvanticity to stimulate robust CD8 ⁺ T cell responses, including mucosal immunity [34]. Although they designed the gp96-Ig fusion protein, our study expands on the concept by linking CRT to possibly more increase the tumor immune response and antigen presentation.

Strong binding affinities were found, particularly for TLR2, TLR4, CD90, SREC-1, and LOX-1, suggesting innate and adaptive immune activation [35,36]. The gp96-CRT-E7 orientation was selected as final vaccine candidate due to its superior docking scores with TLRs and endocytic receptors, such as LOX-1 and SREC-1. This suggests that the N-terminal locating of gp96 might allow initial receptor engagement, whereas the central Calreticulin location adjusts following interactions a hypothesis supported by the strong performance of constructs with gp96 at the N-terminus (gp96-CRT-E7 and gp96-E7-CRT) compared to those with CRT at the N-terminus. The superior docking of gp96-CRT-E7 with TLR2 and TLR4 is predominantly considerable, as these receptors begins signaling cascades that result in the activation of DCs and generation of pro-inflammatory cytokines [35,37]. Strong interactions with SREC-1 and LOX-1 receptors implicated in HSP-mediated antigen cross-presentation also point to increased potential for guiding antigens into MHC class I pathways, which would boost CTL responses [38–40].

Molecular dynamics simulations provided critical validation of the predicted interactions. All gp96-CRT-E7/receptor complexes maintained stable conformations throughout 50 ns simulations, with RMSD values <0.55 nm and Rg fluctuations < 2 Å. The greater stability observed with SREC-1 and TLR2 compared to LOX-1 and TLR4 may reflect differential binding kinetics that could influence immune activation. Additionally, the full-length gp96-CRT-E7 construct was included for comparison with the multi-epitope construct in subsequent bioinformatics analyses. Physicochemical evaluation indicated that the multiepitope gp96-CRT-E7 construct (18.574 kDa, pI 5.41, and instability index 24.16) was more stable and antigenic than the full-length sequence of gp96-CRT-E7 construct (152.21 kDa, pI 4.54, and instability index 44.88). Both constructs were hydrophilic, and possessed half-lives of more than 10 hours in *E. coli* as well as more than 30 hours in mammalian cells. Secondary structure analysis provided evidence of a compact, helix-rich for gp96-CRT-E7 multiepitope construct that enhanced stability as compared to the full-sequence construct. The absence of disulfide bonds in the multiepitope construct simplifies proper folding in prokaryotic expression systems. Importantly, despite its smaller size, the multiepitope construct retained strong predicted antigenicity and immunogenicity, with multiple linear and conformational B-cell epitopes suggesting potential for humoral immunity.

Successful expression of the multiepitope gp96-CRT-E7 fusion peptide in *E. coli* Rosetta at 18°C, confirmed the practical feasibility of producing this candidate for future studies. Expression under native conditions is advantageous, as it preserves native conformation and simplifies purification. *In vivo* validation study examining its therapeutic potential is in progress, and this construct will be utilized for animal immunization in future studies.

While previous HSP-based HPV vaccines which have employed full-length HSPs or single HSP fragments showed promise in preclinical models, our dual-adjuvant multiepitope strategy offers several potential advantages: (1) focused immune response on immunodominant epitopes; (2) elimination of irrelevant HSP sequences; (3) smaller construct size facilitating production and (4) potential for synergistic effects from combining two distinct HSP-derived adjuvants. The strong docking scores with multiple immune receptors support this synergistic potential. While our comprehensive *in silico* analyses and expression validation provide strong support for the gp96-CRT-E7 construct and are promising, the transition to in vivo studies will necessitate further refinement of immunization protocols, optimization, and evaluation of safety and efficacy in appropriate animal models before clinical translation should be considered. In addition, no in vitro functional tests such as dendritic cell maturation, antigen presentation, T cell activation, or cytokine release (*e.g.,* IFN-γ, IL-2, TNF-α), have been conducted. Indeed, all information concerning the immunostimulatory activity of the molecule was based on predictions made by immunoinformatics studies rather than any experimental data. On the other hand, the schedule of vaccine administration used in C-ImmSim simulations (three doses administered at days 1, 84, and 100) was chosen according to common practices, yet there is no experimental optimization performed specifically for this construct. Thus, functional in vitro tests involving dendritic cells co-cultured with the multiepitope gp96-CRT-E7 fusion peptide, along with in vivo animal studies, are our next studies to confirm the predicted immunogenic activity.

## 5. Conclusion

In summary, six novel multiepitope constructs employing conserved MHC-I/II epitopes from E7 and adjuvants gp96 and calreticulin were designed. The gp96-CRT-E7 multiepitope construct, with gp96 at the N-terminus followed by CRT and E7 epitopes, exhibited superior binding to key immune receptors, favorable physicochemical properties, and stable complex formation in MD simulations. The multiepitope gp96-CRT-E7 construct had greater Th1-inducing potential than its full-length counterpart through further immunoinformatics analysis. MD simulations revealed that the epitope-based gp96-CRT-E7 construct forms stable and compact complexes with immune receptors, indicating a strong potential for effective immune responses. The construct’s translational potential was confirmed using *in silico* study, and supported through its expression in *E. coli* Rosetta. Our integrated *in silico* and expression validation approach in this study suggests strong promise for HPV16 vaccine development.

## Supporting information

S1 File Supplementary Materials and Methods. A detailed description of the materials and methods used in this study is provided in Supporting Information File 1.
(DOCX)

S2 File Supplementary Tables (1–10. The supplementary tables supporting the findings of this study are provided in Supporting Information File 2.
(DOCX)

S1 Fig Molecular docking between epitopes and MHC class I alleles: (a) Successful peptide-protein docking between HLA-B:0801 and gp96 (378–389) with an interaction similarity score of −921.3, (b) Successful peptide-protein docking between gp96 (378–389) and HLA-B0702 with an interaction similarity score of −811.1, (c) Successful peptide-protein docking between CRT (164–185) and HLA-B0702 with an interaction similarity score of −732.4, and (d) Successful peptide-protein docking between CRT (147–173) and HLA-B0801 with an interaction similarity score of −660.1. (TIF)

S2 Fig A: Predicted 3D structures of the gp96-CRT-E7 multiepitope construct by I-TASSER: (a) with C-score of −1.55, (b) with C-score of −2.25, (c) with C-score of −4.07, (d) with C-score of −4.16, and (e) with C-score of −3.34. B: (a) The structure produced by the Robetta baker Server and improved by GalaxyRefine Server; (b) Analysis of the Ramachandran plot by PROCHECK.; (c) ERRAT error values graph.
(TIF)

S3 Fig Secondary structure study of the multiepitope and full-length gp96-CRT-E7 constructs: (a) gp96-CRT-E7 multiepitope secondary structure, (b) gp96-CRT-E7 full-length secondary structure. (TIF)

S4 Fig Linear and discontinuous B-cell epitopes prediction of the gp96-CRT-E7 multiepitope construct (a, b & c) and the full-length gp96-CRT-E7 construct (d, e & f) using BepiPred: (a) Predicted linear B-cell epitopes; (b) Illustrated example of B-cell epitopes of the gp96-CRT-E7 multiepitope construct on 3D structure as shown in yellow color; (c) Illustrated conformational B-cell epitopes present in the gp96-CRT-E7 multiepitope vaccine construct; The light-yellow spheres show the epitopes; (d) Predicted linear B-cell epitopes; (e) Illustrated example of B-cell epitopes of the full-length gp96-CRT-E7 construct on 3D structure as shown in yellow color containing 12 residues (RPSKEVEEDEYK) with score of 0.636; (f) Illustrated conformational B-cell epitopes present in the full-length gp96-CRT-E7 vaccine construct containing 9 residues (R345, P346, S347, E349, V350, E351, E354 and Y355) with score of 0.649; The light-yellow spheres show the epitopes. (TIF)

S5 Fig a) Normal-mode analysis of the multiepitope gp96-CRT-E7/ LOX-1 and SREC-1 complexes: (D&I) The elastic network model uses springs between atoms, indicated by colored dots for stiffness; (E&J) Covariance matrix shows paired residue mobility motions, i.e., uncorrelated (white), correlated (red), and anti-correlated (blue); (C&H) Eigenvalues; (A&F) Averaged RMS indicated by B-factor; (B&G) Deformability. b) Normal-mode analysis of the multiepitope gp96-CRT-E7/ TLR4 and TLR2 complexes: (D&I) The elastic network model uses springs between atoms, indicated by colored dots for stiffness; (E&J) Covariance matrix shows paired residue mobility motions, i.e., uncorrelated (white), correlated (red), and anti-correlated (blue); (C&H) Eigenvalues; (A&F) Averaged RMS indicated by B-factor; (B&G) Deformability (TIF)

S6 Fig Schematic representation of *in silico* cloning of the gp96-CRT-E7 fusion gene in pET24a (+) prokaryotic expression vector: (a) the multiepitope gp96-CRT-E7 gene cloned into pET24a (+) vector using *Sal*I and *Hind*III restriction enzymes, (b) the full-length gp96-CRT-E7 gene cloned into pET24a (+) vector using *Pae*R71 and *Eco*53kI restriction enzymes. (TIF)

## References

[1] Chabeda A, Yanez RJR, Lamprecht R, Meyers AE, Rybicki EP, Hitzeroth II. Therapeutic vaccines for high-risk HPV-associated diseases. Papillomavirus Res. 2018;5:46–58. doi: 10.1016/j.pvr.2017.12.006 29277575 PMC5887015

[2] Boliukh I, Rombel-Bryzek A, Radecka B. Immunological aspects of heat shock protein functions and their significance in the development of cancer vaccines. Nowotwory Journal of Oncology. 2022;72(3):174–83. doi: 10.5603/njo.a2022.0024

[3] Das JK, Xiong X, Ren X, Yang J-M, Song J. Heat Shock Proteins in Cancer Immunotherapy. J Oncol. 2019;2019:3267207. doi: 10.1155/2019/3267207 31885572 PMC6927063

[4] Regimbeau M, Abrey J, Vautrot V, Causse S, Gobbo J, Garrido C. Heat shock proteins and exosomes in cancer theranostics. Seminars in Cancer Biology. 2022. 10.1016/j.semcancer.2021.07.014 34343652

[5] Corigliano MG, Sander VA, Sánchez López EF, Ramos Duarte VA, Mendoza Morales LF, Angel SO, et al. Heat Shock Proteins 90 kDa: Immunomodulators and Adjuvants in Vaccine Design Against Infectious Diseases. Front Bioeng Biotechnol. 2021;8:622186. doi: 10.3389/fbioe.2020.622186 33553125 PMC7855457

[6] Basu S, Binder RJ, Ramalingam T, Srivastava PK. CD91 is a common receptor for heat shock proteins gp96, hsp90, hsp70, and calreticulin. Immunity. 2001;14(3):303–13. doi: 10.1016/s1074-7613(01)00111-x 11290339

[7] Didenko G, Kruts O, Skivka L, Prylutskyy Y. The effectiveness of antitumor vaccine enriched with a heat shock protein 70. HSP70 in Human Diseases and Disorders. 2018. p. 325–45.

[8] Pawaria S, Binder RJ. CD91-dependent programming of T-helper cell responses following heat shock protein immunization. Nat Commun. 2011;2:521. doi: 10.1038/ncomms1524 22045000 PMC3356570

[9] Wang J, Gao ZP, Qin S, Liu CB, Zou LL. Calreticulin is an effective immunologic adjuvant to tumor-associated antigens. Exp Ther Med. 2017;14(4):3399–406. doi: 10.3892/etm.2017.4989 29042925 PMC5639401

[10] Zhang H, Zheng H, Guo P, Hu L, Wang Z, Wang J, et al. Broadly Protective CD8+ T Cell Immunity to Highly Conserved Epitopes Elicited by Heat Shock Protein gp96-Adjuvanted Influenza Monovalent Split Vaccine. J Virol. 2021;95(12):e00507-21. doi: 10.1128/JVI.00507-21 33827939 PMC8315956

[11] Sher H, Sharif H, Zaheer T, Khan SA, Ali A, Javed H, et al. Employing computational tools to design a multi-epitope vaccine targeting human immunodeficiency virus-1 (HIV-1). BMC Genomics. 2023;24(1):276. doi: 10.1186/s12864-023-09330-4 37226084 PMC10206567

[12] Gomari MM, Rostami N, Faradonbeh DR, Asemaneh HR, Esmailnia G, Arab S, et al. Evaluation of pH change effects on the HSA folding and its drug binding characteristics, a computational biology investigation. Proteins. 2022;90(11):1908–25. doi: 10.1002/prot.26386 35569112

[13] Zong J, Wang C, Liu B, Liu M, Cao Y, Sun X, et al. Human hsp70 and HPV16 oE7 fusion protein vaccine induces an effective antitumor efficacy. Oncol Rep. 2013;30(1):407–12. doi: 10.3892/or.2013.2445 23660931

[14] Chu NR, Wu HB, Wu T, Boux LJ, Siegel MI, Mizzen LA. Immunotherapy of a human papillomavirus (HPV) type 16 E7-expressing tumour by administration of fusion protein comprising Mycobacterium bovis bacille Calmette-Guérin (BCG) hsp65 and HPV16 E7. Clin Exp Immunol. 2000;121(2):216–25. doi: 10.1046/j.1365-2249.2000.01293.x 10931134 PMC1905702

[15] Garza-Morales R, Perez-Trujillo JJ, Martinez-Jaramillo E, Saucedo-Cardenas O, Loera-Arias MJ, Garcia-Garcia A, et al. A DNA Vaccine Encoding SA-4-1BBL Fused to HPV-16 E7 Antigen Has Prophylactic and Therapeutic Efficacy in a Cervical Cancer Mouse Model. Cancers (Basel). 2019;11(1):96. doi: 10.3390/cancers11010096 30650588 PMC6356763

[16] Haręża DA, Wilczyński JR, Paradowska E. Human Papillomaviruses as Infectious Agents in Gynecological Cancers. Oncogenic Properties of Viral Proteins. Int J Mol Sci. 2022;23(3):1818. doi: 10.3390/ijms23031818 35163748 PMC8836588

[17] Vici P, Pizzuti L, Mariani L, Zampa G, Santini D, Di Lauro L, et al. Targeting immune response with therapeutic vaccines in premalignant lesions and cervical cancer: hope or reality from clinical studies. Expert Rev Vaccines. 2016;15(10):1327–36. doi: 10.1080/14760584.2016.1176533 27063030 PMC5152541

[18] Panahi HA, Bolhassani A, Javadi G, Noormohammadi Z. A comprehensive in silico analysis for identification of therapeutic epitopes in HPV16, 18, 31 and 45 oncoproteins. PLoS One. 2018;13(10):e0205933. doi: 10.1371/journal.pone.0205933 30356257 PMC6200245

[19] Kakakhel S, Ahmad A, Mahdi WA, Alshehri S, Aiman S, Begum S, et al. Annotation of Potential Vaccine Targets and Designing of mRNA-Based Multi-Epitope Vaccine against Lumpy Skin Disease Virus via Reverse Vaccinology and Agent-Based Modeling. Bioengineering (Basel). 2023;10(4):430. doi: 10.3390/bioengineering10040430 37106617 PMC10135540

[20] Kumar KM, Anbarasu A, Ramaiah S. Molecular docking and molecular dynamics studies on β-lactamases and penicillin binding proteins. Mol Biosyst. 2014;10(4):891–900. doi: 10.1039/c3mb70537d 24503740

[21] Rahimi M, Taghdir M, Abasi Joozdani F. Dynamozones are the most obvious sign of the evolution of conformational dynamics in HIV-1 protease. Sci Rep. 2023;13(1):14179. doi: 10.1038/s41598-023-40818-x 37648682 PMC10469195

[22] Chabeda A, Yanez RJR, Lamprecht R, Meyers AE, Rybicki EP, Hitzeroth II. Therapeutic vaccines for high-risk HPV-associated diseases. Papillomavirus Res. 2018;5:46–58. doi: 10.1016/j.pvr.2017.12.006 29277575 PMC5887015

[23] Zong J, Wang C, Wang Q, Peng Q, Xu Y, Xie X, et al. HSP70 and modified HPV 16 E7 fusion gene without the addition of a signal peptide gene sequence as a candidate therapeutic tumor vaccine. Oncol Rep. 2013;30(6):3020–6. doi: 10.3892/or.2013.2742 24065282

[24] Einstein MH, Roden RBS, Ferrall L, Akin M, Blomer A, Wu TC, et al. Safety Run-in of Intramuscular pNGVL4a-Sig/E7(detox)/HSP70 DNA and TA-CIN Protein Vaccination as Treatment for HPV16+ ASC-US, ASC-H, or LSIL/CIN1. Cancer Prev Res (Phila). 2023;16(4):219–27. doi: 10.1158/1940-6207.CAPR-22-0413 36607735 PMC10068439

[25] Multhoff G, Pfister K, Gehrmann M, Hantschel M, Gross C, Hafner M, et al. A 14-mer Hsp70 peptide stimulates natural killer (NK) cell activity. Cell Stress Chaperones. 2001;6(4):337–44. doi: 10.1379/1466-1268(2001)006<0337:AMHPSN>2.0.CO;2 11795470 PMC434416

[26] Krupka M, Zachova K, Cahlikova R, Vrbkova J, Novak Z, Sebela M, et al. Endotoxin-minimized HIV-1 p24 fused to murine hsp70 activates dendritic cells, facilitates endocytosis and p24-specific Th1 response in mice. Immunol Lett. 2015;166(1):36–44. doi: 10.1016/j.imlet.2015.05.010 26021827

[27] Hajikhezri Z, Roohvand F, Maleki M, Shahmahmoodi S, Amirzargar AA, Keshavarz A, et al. HCV Core/NS3 Protein Immunization with “N-Terminal Heat Shock gp96 Protein (rNT (gp96))” Induced Strong and Sustained Th1-Type Cytokines in Immunized Mice. Vaccines (Basel). 2021;9(3):215. doi: 10.3390/vaccines9030215 33802466 PMC7999198

[28] Oli AN, Obialor WO, Ifeanyichukwu MO, Odimegwu DC, Okoyeh JN, Emechebe GO, et al. Immunoinformatics and Vaccine Development: An Overview. Immunotargets Ther. 2020;9:13–30. doi: 10.2147/ITT.S241064 32161726 PMC7049754

[29] Bergmann AC, Kyllesbech C, Slibinskas R, Ciplys E, Højrup P, Trier NH, et al. Epitope Mapping of Monoclonal Antibodies to Calreticulin Reveals That Charged Amino Acids Are Essential for Antibody Binding. Antibodies (Basel). 2021;10(3):31. doi: 10.3390/antib10030031 34449535 PMC8395503

[30] Hong C, Qiu X, Li Y, Huang Q, Zhong Z, Zhang Y, et al. Functional analysis of recombinant calreticulin fragment 39-272: implications for immunobiological activities of calreticulin in health and disease. J Immunol. 2010;185(8):4561–9. doi: 10.4049/jimmunol.1000536 20855873

[31] Karkhah A, Saadi M, Nouri HR. In silico analyses of heat shock protein 60 and calreticulin to designing a novel vaccine shifting immune response toward T helper 2 in atherosclerosis. Comput Biol Chem. 2017;67:244–54. 28189968 10.1016/j.compbiolchem.2017.01.011

[32] Wang S, Fan H, Li Y, Zheng H, Li X, Li C, et al. CD133 epitope vaccine with gp96 as adjuvant elicits an antitumor T cell response against leukemia. Sheng Wu Gong Cheng Xue Bao. 2017;33(6):1006–17. doi: 10.13345/j.cjb.160481 28895362

[33] Zheng H, Liu L, Zhang H, Kan F, Wang S, Li Y, et al. Dendritic cells pulsed with placental gp96 promote tumor-reactive immune responses. PLoS One. 2019;14(1):e0211490. doi: 10.1371/journal.pone.0211490 30703157 PMC6354997

[34] Padula L, Fisher E, Strbo N. “All for One and One for All”: The Secreted Heat Shock Protein gp96-Ig Based Vaccines. Cells. 2023;13(1):72. doi: 10.3390/cells13010072 38201276 PMC10778431

[35] Martinsen JT, Gunst JD, Højen JF, Tolstrup M, Søgaard OS. The Use of Toll-Like Receptor Agonists in HIV-1 Cure Strategies. Front Immunol. 2020;11:1112. doi: 10.3389/fimmu.2020.01112 32595636 PMC7300204

[36] Murshid A, Theriault J, Gong J, Calderwood SK. Molecular Chaperone Receptors. Methods Mol Biol. 2018;1709:331–44. doi: 10.1007/978-1-4939-7477-1_24 29177670 PMC6777869

[37] Ogbodo E, Michelangeli F, Williams JHH. Exogenous heat shock proteins HSPA1A and HSPB1 regulate TNF-α, IL-1β and IL-10 secretion from monocytic cells. FEBS Open Bio. 2023;13(10):1922–40. doi: 10.1002/2211-5463.13695 37583307 PMC10549225

[38] Murshid A, Gong J, Calderwood SK. Heat shock protein 90 mediates efficient antigen cross presentation through the scavenger receptor expressed by endothelial cells-I. J Immunol. 2010;185(5):2903–17. doi: 10.4049/jimmunol.0903635 20686127 PMC4109054

[39] Murshid A, Gong J, Calderwood SK. Hsp90-peptide complexes stimulate antigen presentation through the class II pathway after binding scavenger receptor SREC-I. Immunobiology. 2014;219(12):924–31. 25155057 10.1016/j.imbio.2014.08.001 PMC4886339

[40] Murshid A, Gong J, Calderwood SK. The role of heat shock proteins in antigen cross presentation. Front Immunol. 2012;3:63. doi: 10.3389/fimmu.2012.00063 22566944 PMC3342350

