# Supplementary material for: Orientation of Gp96 and Calreticulin T-cell epitopes in a multiepitope HPV16 E7 vaccine construct affects predicted immunostimulatory properties: An in silico and expression validation study
Source: PLoS One. 2026 Jul 24;21(7):e0353860. doi: 10.1371/journal.pone.0353860 (PMC13399336; doi:10.1371/journal.pone.0353860)
Supplement: S1 File — A detailed description of the materials and methods used in this study is provided in Supporting Information File 1. (DOCX) [file pone.0353860.s001.docx]

**Supplementary information**

**Methods**

**Epitope prediction of gp96 and calreticulin**

**Sequence retrieval of gp96 and calreticulin**

We first identified CD4^+^ and CD8^+^ T-cell epitopes in the sequences of human calreticulin and gp96. The reference protein sequences were obtained from the Uniprot (https://www.uniprot.org) platform for gp96 (UniProt: P14625) and calreticulin (UniProt: P27797) in FASTA format.

**MHC class I (CD8^+^ T-cell or CTL) epitope prediction of gp96 and calreticulin**

Cytotoxic T lymphocytes assist in destroying infected cells. Upon finding the epitopes within the groove of the MHC class I molecule, CTLs will identify them as an antigen. In this research, NetMHCpan 4.0 (http://www.cbs.dtu.dk/services/NetMHCpan/) was employed to forecast the binding of linear peptides (8-11 amino acids) to the MHC class I cavity. The threshold for peptide-MHC-I binding affinity was established at the 2nd percentile for weak binders and at the 0.5th percentile for strong binders. Furthermore, NetMHCIIpan 3.2 (http://www.cbs.dtu.dk/services/NetMHCIIpan/) was employed to forecast the linear binding of peptides (14-16 amino acids) to the groove of MHC class II. The threshold (percentile rank) was set at 2% for strong binders and at 10% for weak binders. The chosen peptides from calreticulin or gp96 were validated for their ability to attach to human HLA-I supertypes, the commonly found HLA-I and -II, and HLA alleles found in Iran at a prevalence of 5% or higher. CTL epitopes were chosen for their highest binding affinity to human MHC-I alleles.

**MHC class II (CD4^+^ T cell or HTL) epitope prediction of gp96 and calreticulin**

To predict interactions between HTL epitopes and commonly occurring MHC-II alleles, NetMHCIIpan 4.0 (http://www.cbs.dtu.dk/services/NetMHCIIpan) and IEDB (http://tools.iedb.org/mhcii/) were utilized, employing standard configurations. Due to the necessity of robust HTL epitope binding to MHC-II for CD4^+^ T-cell activation, this study was performed (1). To determine the likely HTL epitopes with the greatest binding affinity to common human MHC-II alleles, prediction methods were utilized.

**MHC-I immunogenicity and processing prediction**

The IEDB Class I Immunogenicity tool (http://tools.iedb.org/immunogenicity/) was used to evaluate the immunogenicity of the selected MHC-I peptides. The peptide-MHC complex immunogenicity is predicted by this technique based on the properties and positions of amino acids (2). The server's standard settings were employed, and MHC-I peptides with the highest binding ranks to different HLAs were selected to evaluate antigen processing along the antigen presentation pathway. The assessment of proteasomal cleavage and TAP transport effectiveness was carried out using the integrated predictor for proteasomal cleavage/TAP transport/MHC class I sourced from the IEDB database (http://tools.iedb.org/processing/). The three main mechanisms in the IEDB combined predictor's MHC-I antigen presentation pathway are protease processing, MHC-I binding, and TAP transport efficacy, which together determine a comprehensive processing score for every epitope.

**Population coverage and conservancy analysis**

The IEDB population coverage tool (http://tools.immuneepitope.org/tools/population/iedb_input) was utilized to calculate the population coverage percentage for each peptide. In this case, the HLA-I and -II alleles that interact with each anticipated peptide were utilized as the inputs for the analysis of population coverage. The epitope conservancy analysis tool available at the IEDB web server (http://tools.immuneepitope.org/tools/conservancy/iedb_input) was utilized to pinpoint a specific peptide sequence among different HPV subtypes to forecast the conserved cross-reactive epitopes.

**Antigenicity, allergenicity and toxicity analysis**

The VaxiJen v2.0 web server (http://www.ddg-pharmfac.net/vaxijen/VaxiJen/VaxiJen.html), with default settings, was used to examine the antigenicity of epitopes.
The AllergenFP v.1.0 (https:// ddg-pharmfac.net/AllergenFP/) and ToxinPred (<https://webs.iiitd.edu.in/raghava/toxinpred/>) web servers, with default settings, were utilized to evaluate the potential allergenicity and toxicity of the chosen epitopes, respectively.

**Prediction of cytokine production**

The servers IL10Pred (https://webs.iiitd.edu.in/raghava/il10pred/), IL4Pred (https://webs.iiitd.edu.in/raghava/il4pred/), and IFNepitope (https://webs.iiitd.edu.in/raghava/ifnepitope/predict.php) were utilized with default parameters to evaluate the capacity of the selected HTL epitopes in stimulating cytokines including interleukin (IL)-10, IL-4, and interferon-gamma (IFN-γ).

**Prediction of antibody-specific epitopes**

Antibodies can neutralize and eliminate infectious and potentially harmful pathogens (3). The IgPred (https://webs.iiitd.edu.in/raghava/igpred/pep-vari-pred.html) was performed to predict the epitopes that lead to the production of distinct antibodies.

**Screening of linear B-cell epitopes**

B-cell epitopes that can enhance humoral immunity and stimulate antibody production need to be researched for creating multiepitope vaccines. Overlapping analysis may effectively reveal authentic epitopes, despite the fact that B-cell epitope prediction approaches yield unreliable outcomes. This study utilized the BepiPred (https://services.healthtech.dtu.dk/service.php?BepiPred-2.0) and IEDB-based linear epitope prediction (http://tools.iedb.org/bcell/) web servers to assess whether the optimal T-cell epitopes could be classified as B-cell epitopes. In this context, the Random Forest method is applied to identify epitopes from crystal structures. Utilizing the default settings (0.5 as the standard value), we forecasted both residues and epitopes simultaneously at the same location. In locations with B-cell epitopes, the value surpasses the threshold.

**Peptide-MHC flexible molecular docking analysis**

The ClusPro 2.0 (https://cluspro.bu.edu) server) was utilized to estimate the formation of MHC-peptide complexes and forecast the binding interaction of the best-selected peptide with MHC alleles. Each chosen epitope with human MHC alleles underwent a separate peptide-protein flexible molecular docking investigation. Furthermore, the RCSB database (https://www.rcsb.org) provided the PDB files for MHC alleles. For MHC class I, the PDB IDs were 1OGT (HLA-B27:05), 5HGA (HLA-A24:02), 4UQ3 (HLA-A02:01), 3RL2 (HLA-A03:01, 3LKN (HLA-B35:01), 1X7Q (HLA-A11:01), 3SPV (HLAB08:01), and 5EO1 (HLA-B07:02). For MHC class II, they were 4AH2 (HLADRB1:0101), 2Q6W (HLA-DRB1:0301), 5LAX (HLA-DRB1:0401), 6CPL (HLA-DRB1:1101), and 1H15 (HLA-DRB5:0101).

**Multiepitope construct design using immunoinformatics analyses: Linkage of the gp96 or calreticulin epitopes to HPV E7 epitopes**

After performing a wide range of analyses on the predicted epitopes of gp96 and calreticulin proteins, including T-cell epitope prediction, cytokine production profiling, toxicity, allergenicity and antigenicity evaluations, proteasome processing and TAP transport prediction, population coverage analyses, and docking analysis, specific immunogenic epitopes were selected. Moreover, the E7 epitopes of HPV16 used in this study were selected based on our previous work, which identified and introduced E7-specific epitopes from HPV types 16, 18, 31, and 45 (4) and were linked to the CTL and HTL epitopes of gp96 or calreticulin with higher scores from *in silico* analyses. Epitopes were linked in tandem by AAY proteolytic linker using the SnappGene®3.2.1 tool to design six multiepitope constructs in different orientations (*e.g.,* gp96-E7-calreticulin, E7-gp96-calreticulin, gp96-calreticulin-E7, calreticulin-E7-gp96, calreticulin-gp96-E7, E7-calreticulin-gp96) to optimize immunogenicity. These designed multiepitope constructs were evaluated for peptide-protein interactions using molecular docking tools after secondary and tertiary structure prediction and refinement.

**Selection of final multiepitope construct**

After refinement of predicted 3D structures, molecular docking analysis was performed for six designed constructs. The final multiepitope candidate construct was selected based on the superior binding affinity and interface stability with toll-like receptors. This construct was then selected for further comparative analyses. The full-length protein sequence harboring the gp96, calreticulin and E7 was used to compare with the same final multiepitope construct in subsequent bioinformatics analyses. The *in silico* analyses including physicochemical property evaluation, linear and discontinues B-cell epitope prediction, predicting disulfide-bonding state were performed on both the final multiepitope construct and its corresponding full-length construct.

**Refinement and tertiary structure prediction**

The tertiary structure of six multiepitope arrangements was predicted utilizing the Iterative Threading Assemblage Refinement (I-TASSER) online platform (https://zhanglab.ccmb.med.umich.edu/I-TASSER/). The tertiary structure of a protein determines its biological function. I-TASSER employs iterative structure assembly simulations based on amino acid sequences and diverse string arrangements to create 3D atomic models (5). The anticipated tertiary structures were enhanced using the GalaxyRefine service (http://galaxy.seoklab.org/cgi-bin/submit.cgi?type=improve). This technique rebuilds side chains, performs side-chain repacking, and then stabilizes the entire structure using molecular dynamics simulation. To assess the quality of the expected tertiary structures, the completed improved models were subjected to further evaluation using the ERRAT web server (https://servicesn.mbi.ucla.edu/ERRAT). The overall quality factor (OQF) for non-bonded atomic interactions was assessed using the ERRAT web server. Generally, an OQF above 50% for any particular structure indicates a high-quality model (6).

**Protein-protein docking between toll-like receptors/ specific receptors and designed constructs**

Heat shock proteins have been identified as activators of the innate immune response by interacting with toll-like receptors (TLRs) and initiating a signaling cascade [(7). ClusPro 2.0 was employed for docking toll-like receptors with multiepitope constructs (https://cluspro.bu.edu). Six designed constructs were given their final refined tertiary structures as ligands for TLR-2, TLR-3, TLR-4, TLR-5, TLR-8, and TLR-9 in order to perform this protein-protein docking. Using the RCSB database (https://www.rcsb.org), the TLRs' PDB files (TLR-2 PDB ID: 2Z7X, TLR-3 PDB ID: 1ZIW, TLR-4 PDB ID: 3FXI, TLR-5 PDB ID: 3J0A, TLR-8 PDB ID: 3W3G, and TLR-9 PDB ID: 3WPB) were recovered. Software called ChimeraX-1.1 was also used to view the docking findings. In addition to antigens, heat shock proteins can initiate an adaptive immune response by interacting with several endocytic receptors, such as CD91, lectin-like oxidized low-density lipoprotein receptor-1 (LOX-1), clever-1, scavenger receptor expressed by endothelial cells-1 (SREC-1), and CD14 [(8, 9). Therefore, we evaluated how effectively the main receptors docked with the suggested constructs. The PDB data on the principal human receptors was first acquired from the Protein Data Bank (http://www.rcsb.org/). The ClusPro service (https://cluspro.bu.edu/) was utilized to identify the optimal orientation between a construct (as a ligand) and a receptor. The optimal orientation and matching candidate construct with the most desired docking score was ultimately selected for further *in silico* and *in vitro* investigations.

**Comparative immunoinformatics analysis between final multiepitope construct and full length construct**

**Secondary structure prediction**

The secondary structures of final multiepitope construct and its corresponding full-length sequence were predicted using RaptorX (http://raptorx2.uchicago.edu/StructurePropertyPred/predict/) and PSIPRED 4.0 (http://bioinf.cs.ucl.ac.uk/psipred/). A rigorous cross-approval method was used by the free prediction tool PSIPRED 4.0 to get an average Q3 score of 81.6%.

**Physicochemical features**

The ProtParam tools (https://web.expasy.org/protparam/) were utilized to assess the physicochemical properties of the target multiepitope and complete length structures, such as molecular weight, positively and negatively charged residues, along with theoretical pI. Additionally, the solubility of constructs was forecasted utilizing the Protein-Sol web service (https://protein-sol.manchester.ac.uk/). We utilized Aller-TOP2.0 (http://www.ddg-pharmfac.net/AllerTOP/) to assess allergenicity. AllerTOP is a server that predicts allergens *in silico* utilizing the main physicochemical properties of proteins (10).

**Linear and discontinues B-cell epitope prediction**

In order to create multiepitope vaccines, B-cell epitopes are predicted because they have the ability to stimulate humoral immunity and antibody production (11).The IEDB-based linear epitope prediction (<http://tools.iedb.org/bcell/>) web server was utilized for the designed multiepitope and full length constructs to ascertain whether the best T-cell epitopes qualify as B-cell epitopes or not. This program uses crystal structures to detect epitopes using the Random Forest technique. Using the default settings (*i.e.,* 0.5 as the default value), we predicted the residues within the same area as well as the epitopes. The value is higher than the threshold value in regions that contain B-cell epitopes. Furthermore, antibodies are able to identify conformational epitopes in 3D models. Each approved 3D model was assessed by the Ellipro server (https://tools.iedb.org/ellipro/) using the default epitope prediction settings in order to identify non-linear B-cell epitopes (12).

**Predicting disulfide-bonding state**

The DIpro scratch protein predictor service (http://scratch.proteomics.ics.uci.edu/) was utilized in our investigation to forecast the development of disulfide bonds. Disulfide bridges are among the many protein structural properties that may be predicted using this method. The DIpro scratch protein predictor can identify disulfide bonds with 85% precision and a 90% recall rate, assess the number of disulfide bonds, and forecast the bonding status of each cysteine residue along with the associated bonds [(13). Support vector machines (SVMs) and 2D-RNN architectures are used in the DIpro predictor to distinguish between proteins with and without disulfide bonds. Furthermore, it pairs the cysteine residues using graph matching techniques (14).

***In silico* cloning of the designed multiepitope and full length constructs**

The codon usage in prokaryotic organisms is tailored to enhance the expression rate of a vaccine component. Herein, this optimization was performed using the Java Codon Adaptation Tool (JCat) (http://www.jcat.de/CAICalculation.jsp). The synonymous codon usage bias for the vaccine component's DNA sequence was measured using the Codon Adaptation Index (CAI) (15). The SnapGene 3.2.1 program was also utilized to confirm the sequenced constructions, identify restriction enzyme sites, simulate cloning, and insert the changed codon sequences into the expression pET-24a (+) vector.

**Confirmation of final multiepitope construct**

The bioinformatics analyses were completed by performing molecular dynamics simulations and immune simulation on the final multiepitope construct according to the molecular docking results with toll-like receptors and specific receptors.

**Immune simulation of final multiepitope construct**

The C-ImmSim service (https://kraken.iac.rm.cnr.it/C-IMMSIM/) was used to investigate the immunogenicity and immune response profile of peptide vaccines (16). The Celada-Seiden model is used by the C-ImmSim server, an *in silico* immune simulation technique, to forecast the host's immune response profile after exposure to the vaccine constructs. In the simulation, homozygous host haplotypes HLA-A0101, HLA-A0201, HLA-B0702, HLA-DRB10101, and HLA-DRB1*0401 were used, and three doses of the vaccine were given at 1, 84, and 100 intervals without the use of LPS. The parameters were left at their default settings with a random seed of 12,345 and simulation stages of 10 and 1000, respectively.

**Normal mode analysis of final multiepitope construct**

The iMODS server (http://imods.chaconlab.org) was employed to evaluate the movement and stability of the multiepitope construct interacting with the gp96 and calreticulin receptor. The iMODS server, an essential resource for molecular dynamics simulations, is used to conduct normal mode analysis (NMA) in internal coordinates on protein and nucleic acid structures. The server offers various features that assist researchers in comprehending the collective dynamics, stability, adaptability, and versatility of proteins and nucleic acids. This server was selected due to its enhanced speed and effectiveness compared to other MD simulation techniques (17). To evaluate the rigidity and flexibility of the structure, as well as the associated, independent, and oppositional movements in dynamic regions, the server offers deformability, B-factors, eigenvalues, covariance maps, and elastic networks [(18). The ability of a molecule to modify at each residue is referred to called deformability. B-factors derived from NMA are used to determine the mobility of proteins and other macromolecules. The rigidity of motion, which is directly related to the deformation of the structure, is shown by the eigenvalue. The structure of the macromolecule will deform more readily if the number is less. The covariance matrix generates the movements in the protein and displays the correlation between residue pairs. The elastic network detects atom pairs that are connected by springs (19).

**Molecular Dynamics (MD) simulation of final multiepitope construct and receptors**

Gromacs software v.2021.5 was used to dock the vaccine constructs with the receptors and run molecular dynamics simulations on the best-formed complexes for 50 ns. Complex biomolecular systems may be efficiently and concurrently simulated using this program (20). We used the CHARMM-36 force field parameters for topology creation (21) to generate the complexes containing TLR2, TLR4, LOX-1, and SREC-1 receptors. The complexes were positioned one nanometer from the box's boundaries inside a simulated box. The systems were solvated employing the TIP3P water model, and the addition of salt and chloride ions neutralized the total charges.
Before the equilibration phase, energy was refined using the steepest descent technique. The equilibration stage occurred in the NPT ensemble for one nanosecond at a temperature of 298 K and a pressure of 1 bar, employing a Berendsen thermostat and barostat. Every system was simulated for one nanosecond at 300 K under controlled volume and temperature (NVT) settings using a modified Berendsen thermostat (22). Subsequently, the systems were modeled under constant volume and constant pressure (NPT) conditions utilizing a Berendsen barostat to maintain a pressure of 1 atm. The Particle Mesh Ewald (PME) method was employed to calculate long-range electrostatic interactions up to a threshold distance of 1.0 nm (23). The trajectories were utilized for additional analysis after the elimination of periodic boundary conditions (PBC). Several properties, such as the radius of gyration (Rg) between the ligand and receptor, root-mean-square deviation (RMSD), and root-mean-square fluctuation (RMSF), were utilized to assess the conformational stability of the complexes.

**Experimental validation**

**Preparation of the gp96-CRT-E7 fusion DNA construct**

Initially, an amino acid reverse translation program (http://www.bioinformatics.org/sms2/rev_trans.html) was used to extract the nucleotide sequence of the gp96-CRT-E7 multiepitope peptide construct, and the restriction enzyme sites were identified for the cloning procedure. Next, Shine Gene Company (China) created the gp96-CRT-E7 DNA construct in a pUC57 cloning vector. *Sal*I and *Hind*III restriction enzymes were then used to digest the pUC57-gp96-CRT-E7 and the pET-24a (+) expression vector. A Thermo Fisher gel purification kit was used to remove the linearized vector and insert from an agarose gel. After ligating the insert and vector with T4 DNA ligase (Fermentas), the ligation product was transformed into the *E. coli* DH5α strain.

T7 promoter primers were used for colony PCR following the subcloning of the insert into pET-24a (+). A Qiagen DNA extraction Miniprep Kit was used to extract plasmids from the positive colonies. Using 1% agarose gel electrophoresis and restriction enzyme digestion, the recombinant plasmid's integrity was verified. Lastly, a NanoDrop spectrophotometer was used to test the recombinant plasmid's concentration and purity.

**Expression of the recombinant gp96-CRT-E7 multiepitope peptide**

The recombinant multiepitope peptide was expressed by transforming the BL21 (DE3) and Rosetta *E. coli* strains with the recombinant pET-24a (+)-gp96-CRT-E7. From each strain, one recombinant kanamycin-resistant colony was chosen, and it was cultivated in LB/kanamycin medium (Sigma) while being shaken at 150 rpm for the entire night at 37°C. Each culture received a kanamycin inoculation to an optical density (OD_600_) of 0.6–0.7 in the new 2xYT medium (Peptone 1.6%, Yeast 1%, NaCl 0.5%). The addition of 0.5 mM isopropyl thiogalactopyranoside (IPTG, Sigma) was used to induce expression. The incubation period was optimized at two temperatures, 18ºC and 37ºC, and lasted for 4 and 24 hours following IPTG induction. The bacterial pellet was harvested, and the expression of multiepitope peptide was evaluated by 12.5% sodium dodecyl sulfate-polyacrylamide gel electrophoresis (SDS-PAGE). We used an anti-His tag antibody (Abcam, dilution 1:10,000 *v/v*) in a western blot analysis to validate the expression of multiepitope peptide.

**Solubility assessment of the recombinant gp96-CRT-E7 multiepitope peptide**

The solubility of the recombinant gp96-CRT-E7 multiepitope peptide was assessed using overnight *E. coli* cultures harvested by centrifugation. The cell pellets were resuspended in lysis buffer (50 mM sodium phosphate, 20 mM imidazole, 300 mM sodium chloride, 10% glycerol, pH 8.0) supplemented with phenylmethylsulfonyl fluoride (PMSF) and lysozyme. Following a 30-minute incubation on ice and sonication for 10 minutes at a 50% duty cycle, the suspensions were centrifuged at 14,000 rpm for 30 minutes at 4°C. The supernatant (soluble fraction) was collected, and the pellet (insoluble fraction) was resuspended in 1.5 mL of 8 M urea. Both fractions were analyzed by SDS-PAGE.

**Purification of the recombinant multiepitope peptide**

Affinity chromatography was used to purify the recombinant gp96-CRT-E7 multiepitope peptide using HisPur Ni-NTA resin (Thermo Fisher Scientific) under native conditions specified by the manufacturer. Purified multiepitope peptide was then dialyzed against phosphate buffer saline (PBS) 1X using a dialysis membrane (10 kDa, Thermo Fisher Scientific). Its purity and concentration were then assessed using the NanoDrop spectrophotometer and Bradford kit. The LAL test confirmed that the level of lipopolysaccharide (LPS) contamination was less than 0.5 EU/mg (QCL-1000). The pure multiepitope peptide was stored at -70°C for long-term preservation.

1. Vyasamneni R, Kohler V, Karki B, Mahimkar G, Esaulova E, McGee J, et al. A universal MHCII technology platform to characterize antigen-specific CD4(+) T cells. Cell Rep Methods. 2023;3(1):100388.

2. Vita R, Overton JA, Greenbaum JA, Ponomarenko J, Clark JD, Cantrell JR, et al. The immune epitope database (IEDB) 3.0. Nucleic acids research. 2015;43(D1):D405-D12.

3. Sebina I, Pepper M. Humoral immune responses to infection: common mechanisms and unique strategies to combat pathogen immune evasion tactics. Curr Opin Immunol. 2018;51:46-54.

4. Panahi HA, Bolhassani A, Javadi G, Noormohammadi Z. A comprehensive in silico analysis for identification of therapeutic epitopes in HPV16, 18, 31 and 45 oncoproteins. PLoS One. 2018;13(10):e0205933.

5. Yang J, Zhang Y. I-TASSER server: new development for protein structure and function predictions. Nucleic Acids Res. 2015;43(W1):W174-81.

6. Colovos C, Yeates TO. Verification of protein structures: patterns of nonbonded atomic interactions. Protein science. 1993;2(9):1511-9.

7. Ogbodo E, Michelangeli F, Williams JHH. Exogenous heat shock proteins HSPA1A and HSPB1 regulate TNF-α, IL-1β and IL-10 secretion from monocytic cells. FEBS Open Bio. 2023;13(10):1922-40.

8. Pawaria S, Binder RJ. CD91-dependent programming of T-helper cell responses following heat shock protein immunization. Nature communications. 2011;2(1):521.

9. Didenko G, Kruts O, Skivka L, Prylutskyy Y. The effectiveness of antitumor vaccine enriched with a heat shock protein 70. HSP70 in Human Diseases and Disorders. 2018:325-45.

10. Dimitrov I, Bangov I, Flower DR, Doytchinova I. AllerTOP v.2--a server for in silico prediction of allergens. J Mol Model. 2014;20(6):2278.

11. Ahmad TA, Eweida AE, Sheweita SA. B-cell epitope mapping for the design of vaccines and effective diagnostics. Trials in Vaccinology. 2016;5:71-83.

12. Larsen JE, Lund O, Nielsen M. Improved method for predicting linear B-cell epitopes. Immunome Res. 2006;2:2.

13. Cheng J, Randall AZ, Sweredoski MJ, Baldi P. SCRATCH: a protein structure and structural feature prediction server. Nucleic Acids Res. 2005;33(Web Server issue):W72-6.

14. Baldi P, Pollastri G. The principled design of large-scale recursive neural network architectures--dag-rnns and the protein structure prediction problem. The Journal of Machine Learning Research. 2003;4:575-602.

15. Grote A, Hiller K, Scheer M, Münch R, Nörtemann B, Hempel DC, Jahn D. JCat: a novel tool to adapt codon usage of a target gene to its potential expression host. Nucleic Acids Res. 2005;33(Web Server issue):W526-31.

16. Rapin N, Lund O, Bernaschi M, Castiglione F. Computational immunology meets bioinformatics: the use of prediction tools for molecular binding in the simulation of the immune system. PLoS One. 2010;5(4):e9862.

17. López-Blanco JR, Aliaga JI, Quintana-Ortí ES, Chacón P. iMODS: internal coordinates normal mode analysis server. Nucleic Acids Res. 2014;42(Web Server issue):W271-6.

18. Lopéz-Blanco JR, Garzón JI, Chacón P. iMod: multipurpose normal mode analysis in internal coordinates. Bioinformatics. 2011;27(20):2843-50.

19. Dubanevics I, McLeish TCB. Optimising Elastic Network Models for Protein Dynamics and Allostery: Spatial and Modal Cut-offs and Backbone Stiffness. Journal of Molecular Biology. 2022;434(17):167696.

20. Ghahremanian S, Rashidi MM, Raeisi K, Toghraie D. Molecular dynamics simulation approach for discovering potential inhibitors against SARS-CoV-2: A structural review. J Mol Liq. 2022;354:118901.

21. Best RB, Zhu X, Shim J, Lopes PE, Mittal J, Feig M, Mackerell AD, Jr. Optimization of the additive CHARMM all-atom protein force field targeting improved sampling of the backbone φ, ψ and side-chain χ(1) and χ(2) dihedral angles. J Chem Theory Comput. 2012;8(9):3257-73.

22. Bussi G, Donadio D, Parrinello M. Canonical sampling through velocity rescaling. J Chem Phys. 2007;126(1):014101.

23. Petersen HG. Accuracy and efficiency of the particle mesh Ewald method. The Journal of chemical physics. 1995;103(9):3668-79.
