# Supplementary material for: Orientation of Gp96 and Calreticulin T-cell epitopes in a multiepitope HPV16 E7 vaccine construct affects predicted immunostimulatory properties: An in silico and expression validation study
Source: PLoS One. 2026 Jul 24;21(7):e0353860. doi: 10.1371/journal.pone.0353860 (PMC13399336; doi:10.1371/journal.pone.0353860)
Supplement: S2 File — The supplementary tables supporting the findings of this study are provided in Supporting Information File 2. (DOCX) [file pone.0353860.s002.docx]

**Supplementary Table 1:** MHC-I processing prediction scores of calreticulin and gp96 CTL epitopes

| **Protein Name** | **Position** | **Epitope Sequence** | **TAP**  **Score*** | **Proteasome**  **Score**** | **MHC score***** | **Processing score****** | **Immunogenicity******* |
| --- | --- | --- | --- | --- | --- | --- | --- |
|  | 0-22 | MLLSVPLLLGLLGLAVAEPAVY | 0.41 | 1.48 | -3.01 | 1.95 | 0.12113 |
|  | 14-27 | AVAEPAVYFKEQFL | 0.88 | 1.23 | -3.33 | 2.25 | 0.14624 |
|  | 66-80 | QTSQDARFYALSASF | 0.65 | 1.43 | -3.00 | 2.16 | -0.14183 |
| Calreticulin | 78-97 | ASFEPFSNKGQTLVVQFTVK | 0.14 | 0.98 | -3.34 | 1.43 | -0.05357 |
|  | 107-129 | GYVKLFPNSLDQTDMHGDSEYNI | 0.42 | 1.36 | -3.33 | 1.79 | -0.47985 |
|  | 147-173 | FNYKGKNVLINKDIRCKDDEFTHLYTL | 0.41 | 1.33 | -3.15 | 1.79 | 0.18658 |
|  | 164-185 | DDEFTHLYTLIVRPDNTYEVKI | 0.65 | 1.42 | -3.00 | 1.71 | 0.49419 |
|  | 223-266 | KIDDPTDSKPEDWDKPEHIPDPDAKKPEDW | 0.09 | 0.74 | -3.93 | 0.75 | -0.18286 |
|  | 260-289 | WEPPVIQNPEYKGEWKPRQIDNPDYKGTWI | 0.41 | 1.03 | -3.49 | 1.47 | 0.10822 |
|  | 295-307 | NPEYSPDPSIYAY | 0.49 | 1.30 | -3.43 | 1.68 | -0.1484 |
|  | 303-318 | SIYAYDNFGVLGLDLW | 0.49 | 1.53 | -3.00 | 2.16 | 0.26107 |
| Gp96 | 88-114 | IINSLYKNKEIFLRELI | 0.75 | 1.09 | -2.71 | 1.97 | -0.15223 |
|  | 192-208 | GQFGVGFYSAFLVADKV | 0.75 | 1.01 | -2.84 | 1.58 | 0.20565 |
|  | 257-273 | YLELDTIKNLVKKYSQFINFPIY | 0.41 | 1.23 | -2.84 | 1.76 | -0.25816 |
|  | 270-285 | YSQFINFPIYVWSSKT | 0.73 | 1.14 | -2.71 | 1.85 | 0.26725 |
|  | 378-389 | GEVTFKSILFVPTSAPRGLFDEY | 0.41 | 1.17 | -2.84 | 1.63 | 0.22158 |
|  | 420-435 | DFHDMMPKYLNFVKGV | 0.75 | 1.14 | -2.84 | 2.02 | -0.56002 |
|  | 561-576 | YEVIYLTEPVDEYCIQ | 0.61 | 1.03 | -3.12 | 1.66 | 0.4455 |
|  | 738-753 | IERMLRLSLNIDPDAK | 0.34 | 1.41 | -2.84 | 1.90 | -0.12696 |

* A higher score indicates a better quality of tap transport efficiency.

** A higher score indicates a better quality of proteasomal cleavage.

*** A lower MHC score generally indicates a stronger binding affinity.

**** A higher processing score suggests that the epitope is more likely to be processed and presented on the cell surface.

***** A higher score indicates a greater probability of eliciting an immune response

**Supplementary Table 2:** Antigenicity, cytotoxicity, allergenicity of the selected CTL epitopes

| *Protein Name* | *Position* | *Epitope Sequence* | *Allergenicity* | *Cytotoxicity* | *Antigenicity**  *0.4>* |
| --- | --- | --- | --- | --- | --- |
| *Calreticulin* | 0-22 | MLLSVPLLLGLLGLAVAEPAVY | non allergen | Non toxin | 0.6634 |
|  | 14-27 | AVAEPAVYFKEQFL | non allergen | Non toxin | 0.4249 |
|  | 66-80 | QTSQDARFYALSASF | non allergen | Non toxin | 0.7843 |
|  | 78-97 | ASFEPFSNKGQTLVVQFTVK | non allergen | Non toxin | 0.8974 |
|  | 107-129 | GYVKLFPNSLDQTDMHGDSEYNI | non allergen | Non toxin | 0.2086 |
|  | 147-173 | FNYKGKNVLINKDIRCKDDEFTHLYTL | non allergen | Non toxin | 0.8997 |
|  | 164-185 | DDEFTHLYTLIVRPDNTYEVKI | non allergen | Non toxin | 0.3379 |
|  | 223-266 | KIDDPTDSKPEDWDKPEHIPDPDAKKPEDW | non allergen | Non toxin | 0.5845 |
|  | 260-289 | WEPPVIQNPEYKGEWKPRQIDNPDYKGTWI | Probable allergen | Non toxin | 1.2627 |
|  | 295-307 | NPEYSPDPSIYAY | non allergen | Non toxin | 0.7937 |
|  | 303-318 | SIYAYDNFGVLGLDLW | Probable allergen | Non toxin | 0.7989 |
| *Gp96* | 88-114 | IINSLYKNKEIFLRELI | Probable allergen | Non toxin | 0.2066 |
|  | 192-208 | GQFGVGFYSAFLVADKV | non allergen | Non toxin | 0.6795 |
|  | 257-273 | YLELDTIKNLVKKYSQFINFPIY | Probable allergen | Non toxin | 0.0693 |
|  | 270-285 | YSQFINFPIYVWSSKT | non allergen | Non toxin | 0.1315 |
|  | 378-389 | GEVTFKSILFVPTSAPRGLFDEY | non allergen | Non toxin | 0.5187 |
|  | 420-435 | DFHDMMPKYLNFVKGV | non allergen | Non toxin | 0.8936 |
|  | 561-576 | YEVIYLTEPVDEYCIQ | Probable allergen | Non toxin | 0.2483 |
|  | 738-753 | IERMLRLSLNIDPDAK | non allergen | Non toxin | 0.6046 |

* Higher rates than threshold indicates high antigenicity of epitope

**Supplementary Table 3:** Antigenicity, cytotoxicity and allergenicity of the selected HTL epitopes

| Protein Name | Position | Epitope  Sequence | Antigenicity*  > 0.4 | Cytotoxicity | Allergenicity |
| --- | --- | --- | --- | --- | --- |
| Gp96 | 257-273 | YLELDTIKNLVKKYSQFINFPIY | -0.0693  Non antigen | Non-Toxin | Probable allergen |
|  | 378-389 | GEVTFKSILFVPTSAPRGLFDEY | 0.5187  antigen | Non-Toxin | non allergen |
|  | 738-753 | IERMLRLSLNIDPDAK | 0.6046  antigen | Non-Toxin | non allergen |
|  | 192-213 | IINSLYKNKEIFLRELI IVTSK | 0.2890  Non antigen | Non-Toxin | non allergen |
|  | 83-98 | NRMMKLIINSLYKNKE | 0.0109  Non antigen | Non-Toxin | non allergen |
|  | 535-549 | QDKIYFMAGSSRKEA | 0.7182  antigen | Non-Toxin | non allergen |
| Calreticulin | 107-129 | GYVKLFPNSLDQTDMHGDSEYNI | 0.2325  Non antigen | Non-Toxin | non allergen |
|  | 147-173 | FNYKGKNVLINKDIRCKDDEFTHLYTL | 0.8997  antigen | Non-Toxin | non allergen |
|  | 164-185 | DDEFTHLYTLIVRPDNTYEVKI | 0.3379  Non antigen | Non-Toxin | non allergen |

* Higher rates than threshold indicates high antigenicity of epitope.

**Supplementary Table 4:** Screening the cytokine secreting epitopes of gp96 and calreticulin

| **Protein Name** | **Position** | **Epitope Sequence** | **IL-10 Production SVM scores** | **IL-10 Induction** | **IL- 4 Production SVM scores** | **IL-4**  **Induction** | **IFN-ɣ production SVM scores** | **IFN-ɣ**  **induction** |
| --- | --- | --- | --- | --- | --- | --- | --- | --- |
| *Gp96* | 257-273 | YLELDTIKNLVKKYSQFINFPIY | 0.627 | Inducer | 0.32 | Inducer | 1.6926805 | Inducer |
|  | 535-549 | QDKIYFMAGSSRKEA | 0.612 | Inducer | 0.33 | Inducer | -4.3526523 | Non-inducer |
|  | 378-389 | GEVTFKSILFVPTSAPRGLFDEY | 0.598 | Inducer | 0.32 | Inducer | 2.0824246 | Inducer |
|  | 738-753 | IERMLRLSLNIDPDAK | 0.572 | Inducer | 0.25 | Inducer | -0.449706 | Non-inducer |
|  | 270-285 | YSQFINFPIYVWSSKT | -0.22 | Non-Inducer | 0.18 | Non-Inducer | -0.187 | Non-Inducer |
|  | 192-213 | IINSLYKNKEIFLRELI IVTSK | 0.733 | Inducer | 0.05 | Non-Inducer | 0.33 | Inducer |
|  | 83-98 | NRMMKLIINSLYKNKE | 0.534 | Inducer | 0.26 | Inducer | -0.35437904 | Non-inducer |
| *Calreticulin* | 107-129 | GYVKLFPNSLDQTDMHGDSEYNI | 0.547 | Inducer | 0.26 | Inducer | 1.6734213 | Inducer |
|  | 147-173 | FNYKGKNVLINKDIRCKDDEFTHLYTL | 0.603 | Inducer | 0.33 | Inducer | 2.8855797 | Inducer |
|  | 164-185 | DDEFTHLYTLIVRPDNTYEVKI | 0.563 | Inducer | 0.11 | Non-Inducer | 1.4752619 | Inducer |

**Supplementary Table 5:** Screening the antibody-specific epitopes of gp96 and calreticulin

| **Protein Name** | **Position** | **Epitope Sequence** | **IgG score** | **IgE score** | **IgA score** | **B-Cell Epitope** |
| --- | --- | --- | --- | --- | --- | --- |
| *Gp96* | 257-273 | YLELDTIKNLVKKYSQFINFPIY | -0.144 | 0.512 | 0.750 | Non-Epitope |
|  | 378-389 | GEVTFKSILFVPTSAPRGLFDEY | 1.126 * | -0.686 | 0.053 | IgG Epitope |
|  | 738-753 | IERMLRLSLNIDPDAK | 0.403 | -0.776 | 0.088 | Non-Epitope |
|  | 88-104 | IINSLYKNKEIFLRELI | 0.356 | -1.050 | 0.087 | Non-Epitope |
|  | 192-208 | GQFGVGFYSAFLVADKV | -0.249 | -0.414 | -0.932 | Non-Epitope |
|  | 270-285 | YSQFINFPIYVWSSKT | -0.141 | 0.536 | 0.293 | Non-Epitope |
|  | 561-576 | YEVIYLTEPVDEYCIQ | 0.376 | 0.165 | 0.268 | Non-Epitope |
|  | 420-435 | DFHDMMPKYLNFVKGV | 0.724 | 0.470 | 0.397 | Non-Epitope |
| *Calreticulin* | 66-80 | QTSQDARFYALSASF | -0.684 | 0.094 | 0.018 | Non-Epitope |
|  | 0-22 | MLLSVPLLLGLLGLAVAEPAVY | -0.057 | -0.902 | -0.519 | Non-Epitope |
|  | 107-129 | GYVKLFPNSLDQTDMHGDSEYNI | -0.198 | 0.541 | 0.593 | Non-Epitope |
|  | 147-173 | FNYKGKNVLINKDIRCKDDEFTHLYTL | 0.507 | -0.698 | 0.638 | Non-Epitope |
|  | 164-185 | DDEFTHLYTLIVRPDNTYEVKI | 0.991 | -0.949 | 0.638 | IgG Epitope |
|  | 14-27 | AVAEPAVYFKEQFL | -0.219 | -0.419 | 0217 | Non-Epitope |
|  | 223-266 | KIDDPTDSKPEDWDKPEHIPDPDAKKPEDW | 1.349 * | -0.470 | 0.546 | IgG Epitope |
|  | 260-289 | WEPPVIQNPEYKGEWKPRQIDNPDYKGTWI | 1.133 * | -0.440 | 0.115 | IgG Epitope |
|  | 295-307 | NPEYSPDPSIYAY | 0.615 | -0.158 | -0.065 | Non-Epitope |
|  | 303-318 | SIYAYDNFGVLGLDLW | -0.333 | -0.373 | 0.471 | Non-Epitope |
|  | 78-97 | ASFEPFSNKGQTLVVQFTVK | -0.215 | -0.004 | 1.108 | IgA Epitope |

**Supplementary Table 6:** B-cell epitopes predicted for the selected gp96 and calreticulin epitopes

| Protein name | Position Epitope Sequence | | Length | Peptide |
| --- | --- | --- | --- | --- |
| *Gp96* | 378-389 | GEVTFKSILFVPTSAPRGLFDEY | 3 | RGL |
| *Calreticulin* | 147-173 | FNYKGKNVLINKDIRCKDDEFTHLYTL | 19 | GKNVLINKDIRCKDDEFTH |
|  | 164-185 | DDEFTHLYTLIVRPDNTYEVKI | 4 | DNTY |

**Supplementary Table 7:** The interaction similarity scores of the identified putative E7 protein, gp96, and calreticulin CTL epitopes and MHC molecules using the Galaxy PepDock flexible docking server

| Epitope/MHC  Allele | HLAB:  2705  1OGT | HLAA:  2402  5HGA | HLAA:  0201  4UQ3 | HLAA:  0301  3RL2 | HLAB:  3501  3LKN | HLAA:  1101  1X7Q | HLAB:  0801  3SPV | HLAB:  0702  5EO1 | HLADRB1:0101  4AH2 | HLA-DRB1:  0301  2Q6W | HLADRB1:0401  5LAX | HLA-DRB1:  1101  6CPL | HLADRB5:  0101  1H15 |
| --- | --- | --- | --- | --- | --- | --- | --- | --- | --- | --- | --- | --- | --- |
| Gp96  192-208 | -741.1 | -935.9 | -730.2 | -814.7 | -718.4 | -542.8 | -797.3 | -690.2 | -854.5 | -885.7 | -725.5 | -725.5 | -725.5 |
| Gp96  378-389 | -805.3 | -821.0 | -865.0 | -797.7 | -788.3 | -870.5 | -921.3 | -811.1 | -900.8 | -1016.6 | -1008.3 | -921.2 | -1211.0 |
| CRT  164-185 | -655.1 | -703.9 | -905.7 | -716.5 | -695.1 | -668.3 | -782.4 | -705.5 | -822.4 | -883.8 | -884.4 | -860.8 | -910.9 |
| CRT  147-173 | -790.1 | -808.7 | -866.2 | -838.5 | -803.7 | -938.1 | -807.6 | -765.3 | -908.7 | -962.7 | -830.8 | -889.8 | -862.7 |
| E7  49–57 | -667.2 | -669.1 | -729.7 | -606.3 | -605.0 | -639.0 | -612.4 | -594.4 | -740.6 | -920.8 | -848.7 | -703.3 | -869.7 |
| E7  43–52 | -667.2 | -669.1 | -729.7 | -606.3 | -605.0 | -639.0 | -612.4 | -594.4 | -740.6 | -920.8 | -848.7 | -703.3 | -869.7 |
| E7  7-21 | -543.6 | -640.2 | -676.9 | -646.4 | -574.7 | -569.2 | -588.8 | -538.6 | -652.1 | -772.8 | -714.3 | -623.1 | -791.8 |
| E7  71–79 | -680.6 | -639.8 | -633.6 | -652.6 | -647.3 | -659.8 | -625.7 | -601.0 | -702.1 | -750.3 | -678.5 | -697.1 | -735.3 |

| Protein  name | Epitope | Location  (length) | Immunogenicity  Score | Population  Coverage | Proteasomal  Cleavage score | TAP  score | Processing  score | Toxicity | Allergenicity | Antigenicity | IFN-γ | B-Cell  Epitope |
| --- | --- | --- | --- | --- | --- | --- | --- | --- | --- | --- | --- | --- |
| HPV E7 | RAHYNIVTF | 49–57 (9) | 0.18 | 99.98% | 1.48 | 1.18 | 2.66 | *Non-toxic* | *Non- Allergen*  *0.73* | *0.7355*  *antigen* | *inducer* | *Non-inducer* |
|  | STHVDIRTL | 71–79 (9) | 0.27 | 99.35% | 1.79 | 0.42 | 2.21 | *Non-toxic* | *Allergen* | *1.9249*  *antigen* | *inducer* | *inducer* |
|  | TLHEYMLDL | 7–15 (9) | -0.05 | 96.26% | 1.17 | 0.37 | 1.53 | *Non-toxic* | *Non- Allergen*  *0.62* | *1.2564*  *antigen* | *inducer* | *inducer* |
|  | GQAEPDRAHY | 43–52 (10) | 0.23 | 49.47% | 1.57 | 1.30 | 2.87 | *Non-toxic* | *Non- Allergen*  *0.63* | *0.6532*  *antigen* | *inducer* | *inducer* |

**Supplementary Table 8:** The properties of predicted E7 epitopes

**Supplementary Table 9:** Predicted linear B-cell epitopes of the multiepitope gp96-CRT-E7 construct

| Position | Peptide | Number of residues | Score |
| --- | --- | --- | --- |
| 10-13 | AFLV | 4 | 0.511 |
| 40-54 | APRGLFDEYAAYDDE | 15 | 0.67 |
| 24-35 | AYGEVTFKSILF | 12 | 0.74 |
| 89-99 | DIRCKDDEFTH | 11 | 0.828 |
| 147-163 | PETTDAAYSTHVDIRTLRPDNT | 17 | 0.869 |
| 113-129 | VTFAAYGQAEPDRAHYN | 17 | 0.606 |
| 64-68 | RPDNT | 5 | 0.687 |

**Supplementary Table 10:** Predicted discontinuous epitopes of the multiepitope gp96-CRT-E7 construct

| Residues | Number of residues | Scores |
| --- | --- | --- |
| A:D89, A:I90, A:R91, A:C92, A:K93, A:D94, A:D95, A:E96, A:F97, A:T98, A:H99 | 11 | 0.828 |
| A:P65, A:D66, A:N67, A:T68, A:V71 | 5 | 0.659 |
| A:S21, A:A23, A:A24, A:Y25, A:G26, A:E27, A:V28, A:T29, A:F30, A:K31, A:S32, A:I33, A:L34, A:F35, A:D46, A:E47, A:Y48, A:A49, A:A50, A:Y51, A:D52, A:D53, A:E54 | 23 | 0.668 |
| A:V113, A:T114, A:F115, A:A116, A:A117, A:Y118, A:G119, A:Q120, A:A121, A:E122, A:P123, A:D124, A:R125, A:A126, A:H127, A:Y128, A:N129, A:P147, A:E148, A:T149, A:T150, A:D151, A:A152, A:A153, A:Y154, A:S155, A:T156, A:H157, A:V158, A:D159, A:I160, A:R161, A:T162, A:L163 | 34 | 0.737 |
| A:Y8, A:A10, A:F11, A:L12, A:V13, A:D15, A:A40, A:P41, A:R42, A:G43, A:L44, A:F45, A:R64 | 13 | 0.638 |
